# Mechanical Remodeling and Mechanosensing after Spinal Cord Injury: From Molecular to Translational Approaches

**DOI:** 10.34133/research.1361

**Published:** 2026-07-10

**Authors:** Jier Ma, Xiumei Tang, Jie Tan, Baoshuai Bai, Sen Guo, Shenghui Shang, Zhaoliang Hou, Yixiao He, Zhengdong Zhang, Wenzhao Wang, Zhijian Wei

**Affiliations:** ^1^ West China School of Medicine, West China Hospital, Sichuan University, Chengdu, Sichuan 610041, China.; ^2^ Department of Orthopedic, Qilu Hospital of Shandong University, Jinan, Shandong 250012, China.; ^3^ State Key Laboratory of Respiratory Health and Multimorbidity, Sichuan University, Chengdu 610041, China.; ^4^ Hubei Key Laboratory of Sports Injury and Precision Therapy, Wuhan Fourth Hospital, Wuhan 430030, China.; ^5^ Department of Plastic Surgery, The Second Affiliated Hospital of Zhejiang University College of Medicine, Hangzhou 310000, China.; ^6^ Department of Pathology, Department of Radiology, Juntendo University Graduate School of Medicine, Tokyo 100-8994, Japan.; ^7^ Department of Orthopedics, The First Affiliated Hospital of Chengdu Medical College, Chengdu 610500, China.; ^8^ School of Biomedical Sciences, The Chinese University of Hong Kong, Hong Kong SAR, 999077, China.

## Abstract

Spinal cord injury triggers a profound, spatiotemporally dynamic remodeling of the local mechanical microenvironment. Acute tissue softening and extracellular matrix degradation give way progressively to chronic fibrotic stiffening and dense matrix cross-linking. This pathological transformation establishes a formidable physical barrier to axonal regeneration while simultaneously disrupting the biomechanical signals that normally guide cellular behavior and maintain tissue homeostasis. Within this evolving niche, diverse cell populations decode altered stiffness and matrix composition through an array of specialized mechanosensitive receptors. The resulting intracellular signaling cascades orchestrate lineage-specific transcriptional and cytoskeletal programs that ultimately govern the delicate equilibrium between endogenous repair and irreversible fibrotic scar consolidation. This review examines the temporal progression and spatial heterogeneity of postinjury mechanical alterations across the spinal parenchyma, delineates the principal mechanotransduction pathways engaged, and critically evaluates emerging therapeutic strategies designed to modulate this aberrant physical landscape and restore a permissive regenerative milieu. We highlight translational advances spanning surgical decompression, mechanically responsive biomaterials engineered for dynamic niche reprogramming, and mechanoprimed cellular therapies. By framing spinal cord injury as a disorder of aberrant mechanobiology, this work advances a conceptual foundation for precision interventions aimed at overcoming regenerative failure through active remodeling of the tissue microenvironment.

## Introduction

Spinal cord injury (SCI) is a severe neurological condition resulting from traumatic or nontraumatic damage to the spinal cord, leading to temporary or permanent impairment of motor, sensory, and autonomic functions below the level of injury [1]. The global burden of SCI remained substantial in 2021, with over 15.4 million prevalent cases worldwide and approximately 574,500 new incident cases [2]. These trends collectively position SCI as a critical and dynamic public health challenge, necessitating continued research into its underlying mechanisms and novel treatment strategies.

The pathophysiology of SCI is classically understood as a 2-phase process. An immediate primary injury characterized by mechanical disruption of neural tissue, vasculature, and cellular membranes, followed by a prolonged secondary injury cascade involving ischemia, excitotoxicity, inflammation, oxidative stress, and eventual glial scarring [3]. This secondary injury cascade is accompanied by profound biomechanical remodeling, in which acute tissue edema and sustained shifts in local pressure converge with extensive extracellular matrix (ECM) remodeling. This remodeling is characterized by the dysregulated deposition of structural proteins such as collagen and fibronectin, alongside inhibitory molecules such as chondroitin sulfate proteoglycans (CSPGs) [1]. Consequently, the mechanical properties of the injured tissue, including its stiffness, compliance, and viscoelasticity, are dramatically altered [4]. The remolded ECM actively generates and transmits diverse mechanical cues, including static and dynamic strain, compression, and shear stress [5], which are detected by specialized mechanosensitive receptors on neural cells, triggering intracellular pathways that critically influence cell survival, activation state, and differentiation potential [6,7].This intersection of mechanical forces and biological responses is broadly termed biomechanics or mechanobiology in the context of SCI. Therefore, the postinjury mechanical microenvironment actively shapes the subsequent phases of degeneration, scar formation, and failed regeneration.

Given the established significance of mechanical signaling throughout the continuum of SCI pathogenesis, this review systematically investigates the interplay between mechanobiological mechanisms and emerging therapeutic avenues. By tracing how the evolving postinjury mechanical landscape is sensed and interpreted by diverse cell populations, this review aims to resolve the causal chain that links pathological tissue remodeling to divergent cellular outcomes and to identify the critical nodes where therapeutic intervention may redirect this trajectory toward repair. Rather than treating matrix stiffening, mechanotransduction, and cellular responses as separate phenomena, this review integrates them into a sequential framework that spans from mechanical perturbation to functional consequence, with deliberate emphasis on the spatiotemporal heterogeneity and cell type specificity that distinguish the SCI niche from other models of tissue fibrosis or central nervous system (CNS) injury. In doing so, this review moves beyond cataloging individual mechanosensitive pathways to examine how multiple receptor systems, downstream effectors, and transcriptional programs converge within specific cellular contexts to govern fate decisions under pathological mechanical loads. The subsequent sections trace this continuum through the spatiotemporal evolution of postinjury tissue mechanics, the mechanosensitive receptor systems that interpret these physical signals, the cell-type-specific responses that collectively govern the regenerative-scarring equilibrium, and the therapeutic strategies designed to remodel this aberrant physical microenvironment toward a proregenerative state.

## The Evolving Mechanical Microenvironment Post-SCI

Following SCI, the mechanical microenvironment undergoes a complex spatiotemporal evolution involving dynamic changes in tissue stiffness, ECM composition, cellular constituents, and molecular regulatory networks. These changes exhibit substantial variations across different phases of injury and distinct anatomical regions, collectively shaping a microenvironment that is either inhibitory or permissive to neural repair (Table 1).

**Table 1.** Spatiotemporal heterogeneity of the mechanical microenvironment after spinal cord injury

Region	Injury model	Species	Measurement method	Time point	Stiffness profile	ECM	Cellular composition
**Fibrotic scar**	Crush (T9, aneurysm clip, 50 g, 30 s)	Rat (Sprague–Dawley, F, 11–12 wk)	Microindentation	8–12 wk	Significant stiffening (dense peripheral rim + looser inner core)	Fibrillar collagens, collagen I, fibronectin	Fibroblasts, pericyte, macrophages, absence of neurons and oligodendrocytes
	Hemisection (T9, microknife)	Mouse (CX3CR1^+^/eGFP, C57BL/6, F)	AFM	1–2 wk			
	Severe contusion (T11, 100 kdyne, 60 s)	Mouse (C57BL/6, F, 8 wk)	AFM	12 wk			
**Glial scar**	Spinal dorsal column crush (C5, 1.5 mm)	Rat (lister hooded, F, 8–12 wk)	AFM	1.5–3 wk	Significant softening	CSPGs, tenascin C, laminin	Reactive astrocytes (GFAP^+^), NG2^+^ oligodendrocyte progenitor cells, microglia
**Gray matter**	Dorsal column crush (C5, forceps, ~1.5 mm depth, 2 × 10 s)	Rat (lister hooded, F, 12 wk)	AFM	1–3 wk	Softening occurs both within and outside visible lesion site, but stiffer than white matter	PNNs around motor neurons exhibit a “loosen–tighten” dynamics	Astrocytes; resident gray matter astrocytes markedly diminish postinjury
**White matter**					Softening remains confined to injury site	ECM-related genes, TGF-β2 up-regulated	Astrocytes; white-matter-derived astrocytes migrate to gray matter (Igfbp2^+^)

AFM, atomic force microscopy; CSPGs, chondroitin sulfate proteoglycans; ECM, extracellular matrix; F, female; GFAP, glial fibrillary acidic protein; Igfbp2, insulin-like growth factor binding protein 2; NG2, neuron-glial antigen 2 (chondroitin sulfate proteoglycan 4); PNNs, perineuronal nets; s, seconds; TGF-β2, transforming growth factor-β2; eGFP, enhanced green fluorescent protein.

### Temporal dynamics

**Fig. 1.**
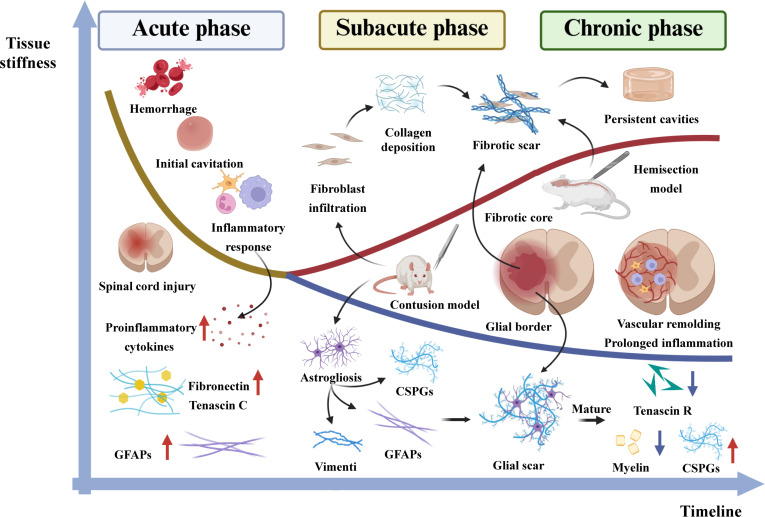
Spatiotemporal dynamics of the mechanical microenvironment after spinal cord injury. The mechanical microenvironment evolves through 3 distinct phases postinjury. Acute phase: Both the lesion core and surrounding tissue exhibit convergent tissue softening, driven by hemorrhage, cavitation, and inflammatory infiltration, with up-regulation of proinflammatory cytokines, fibronectin, tenascin C, and glial fibrillary acidic protein (GFAP). Subacute phase: Tissue stiffness begins to exhibit regional divergence. In crush or hemisection models, the fibrotic core undergoes progressive stiffening due to fibroblast infiltration and fibrillar collagen deposition, accompanied by cavity expansion; in contrast, the glial scar shows persistent low stiffness in the crush model, correlating with reactive astrogliosis and accumulation of vimentin, chondroitin sulfate proteoglycans (CSPGs), and GFAP. Chronic phase: Both fibrotic and glial scars mature, with persistent cavity formation, down-regulation of structural extracellular matrix proteins, such as tenascin R and myelin, and sustained accumulation of CSPGs. Prolonged inflammation and concurrent vascular remodeling occur throughout all phases. (Created with BioRender.com.)

The evolution of the mechanical microenvironment postinjury is a continuum, broadly divisible into acute, subacute, and chronic phases, each characterized by distinct pathological, pathophysiological, and molecular features (Fig. 1). Importantly, the reported stiffness trends are highly dependent on experimental context, including the choice of injury model, the spatial resolution of the measurement technique, and the specific region sampled, as elaborated below.

#### Acute phase (hours to days postinjury)

The hallmark of the acute phase is tissue softening [8]. In a rat thoracic crush injury model, microindentation testing revealed that the elastic modulus of the injury region dropped sharply from a baseline of approximately 9.9 kPa in uninjured tissue to a median of 5.8 kPa at 1 d and further to 3.3 kPa at 3 d postinjury, representing a more than 2-fold reduction relative to uninjured controls [8]. Concurrently, the drop in peak force, an indicator of tissue viscosity, increased from a baseline of approximately 57% in uninjured tissue to 80% at 3 d postinjury, indicating that the acutely injured cord becomes not only softer but also markedly more viscous [8]. During the early postinjury period, disruption of the blood–spinal cord barrier, infiltration of inflammatory cells, and edema lead to ECM degradation and an increase in tissue compliance, resulting in decreased stiffness [9,10]. Pathologically, this phase is marked by hemorrhage, initial cavitation, and the peak of the acute inflammatory response [10]. Cellularly, neurons undergo “transcriptional shock” with marked down-regulation of global gene expression, while astrocytes, oligodendrocyte progenitor cells, and microglia initiate mitotic activation [9]. At the molecular level, there is a rapid up-regulation of proinflammatory cytokines, specific ECM components such as fibronectin and tenascin C, and early glial markers including glial fibrillary acidic protein (GFAP) [11].

#### Subacute phase (days to weeks postinjury)

This phase represents a critical window for the transition of mechanical properties and scar formation. Tissue stiffness begins to diverge. On one hand, softening may persist or even intensify, particularly within the glial scar regions. Atomic force microscopy (AFM) indentation of a rat spinal cord crush injury model revealed significant tissue softening within the lesion site at 8 d and 1.5 weeks postinjury [4]. This correlates with reactive astrogliosis, up-regulation of vimentin and GFAP, and the accumulation of hydrated proteoglycans such as CSPGs [4,9]. On the other hand, the fibrotic process is initiated. Deposition of fibrillar collagens begins, leading to progressive hardening of the injury core [8,12]. Microindentation testing in a rat thoracic contusion model demonstrated that the elastic modulus of the injury region progressively recovered from its acute nadir of approximately 3.3 kPa at 3 d postinjury to 8.1 kPa by 2 weeks and returned to near-baseline levels of approximately 10 kPa by 4 weeks [8]. In addition, AFM force spectroscopy in a mouse thoracic hemisection model revealed that the elastic modulus of the injury site progressively increased from approximately 7 ± 4 kPa at 72 h postinjury to 54 ± 32 kPa by 2 weeks, correlating with increased collagen fiber density and tortuosity as evidenced by second-harmonic generation imaging [13]. Pathologically, the glial and fibrotic scars consolidate, and cystic cavities expand [9]. Angiogenic responses peak around day 7, with basement membrane components laminin providing a temporary permissive scaffold for axonal growth [12]. Cellularly, neuronal transcriptional activity gradually recovers [9]. Notably, a second wave of microglial activation emerges at day 14 postinjury, coinciding temporally with a secondary decline in neuronal populations, suggesting a subsequent round of attack [9]. Molecularly, CSPGs reach peak expression, contributing to an inhibitory microenvironment [14].

#### Chronic phase (weeks to months postinjury)

The chronic phase is characterized by persistent tissue remodeling and stabilization of mechanical properties. The ultimate trend in stiffness varies across studies, likely depending on the injury model and assessment methodology. Some studies report significant tissue stiffening due to the formation of a dense, cross-linked fibrotic scar [8,15]. Microindentation testing in a rat contusion model revealed that by 8 weeks postinjury, the elastic modulus of the injury region had risen to a median of 12.9 kPa and remained elevated at 12 weeks (13.7 kPa), an approximately 4-fold increase over the acute nadir at 3 d postinjury [8]. Similarly, rheological and indentation studies in a mouse contusion model have shown that chronic fibrotic scar stiffness reaches 5 to 10 kPa at 8 weeks postinjury, compared to 0.3 to 0.8 kPa in uninjured tissue [15]. In contrast, others indicate that the mature glial scar core, devoid of collagen I but rich in CSPGs, remains relatively soft [4]. AFM measurements in a rat spinal cord crush injury model demonstrated that significant tissue softening within the lesion site persisted at 3 weeks postinjury, indicating that glial scar softening extends into the subacute-to-chronic transition [4]. These divergent findings are reconciled by differences in injury model, sampling region, and measurement technique. Measurements targeting the collagen-rich fibrotic scar in contusion models, whether by microindentation or AFM, reveal chronic tissue stiffening; in contrast, AFM measurements specifically targeted to the CSPG-rich glial scar in crush injuries resolve microscale tissue softening [4,8,15]. The chronic SCI lesion is thus a mechanically composite structure rather than a uniformly stiff or soft entity.

Pathologically, mature scars, persistent cystic cavities, and dense nuclear packing are established [16]. Chronic vascular remodeling and sustained low-grade inflammation indicate prolonged microenvironmental instability. Cellularly, microglia have not returned to their uninjured state and exhibit elevated expression of Alzheimer’s disease-associated genes, such as *Apoe*, *Spp1*, and *Apoc1*, indicating a sustained adverse immune microenvironment [9]. Furthermore, 3 distinct astrocyte subtypes present in the uninjured spinal cord converge into a single reactive state after injury, forming interconnected reticular structures [9]. What’s more, tip cells serve as the primary source of the proangiogenic factor Plgf (placental growth factor) and the vessel-destabilizing factor Angpt-2 (angiopoietin 2), whereas astrocytes subsequently promote vessel stabilization through Angpt-1 expression, collectively revealing a dynamic transition from angiogenic sprouting to vascular maturation after SCI [17]. Molecularly, expression of some structural ECM proteins, including tenascin R and myelin, may be down-regulated, while inhibitory CSPGs persist [4].

### Spatial heterogeneity

Mechanical changes post-SCI are not uniform but exhibit a gradient-like spatial heterogeneity radiating from the epicenter toward rostral, caudal, and surrounding tissues.

#### Lesion core

The lesion core undergoes the most dramatic alterations. In the chronic phase, a fibrotic scar core typically forms, rich in fibrillar collagens, leading to significant stiffening. This scar exhibits a heterogeneous architecture, comprising a dense peripheral rim of collagen-producing cells that apposes the astroglial scar and encloses a looser inner core [18]. The dense outer rim creates a physically low-porosity barrier that severely impedes cell infiltration and axonal regeneration [19]. Cellularly, this region is dominated by fibroblasts derived from platelet-derived growth factor receptor β-positive type A pericytes that detach from the vascular wall, proliferate, and express α-smooth muscle actin [20], as well as macrophages whose recruitment is mediated by a proliferation inducing ligand via up-regulation of tumor necrosis factor-α and C–C motif chemokine ligand 2 [21], with a notable absence of neurons and oligodendrocytes [22]. The molecular environment of the fibrotic scar is highly inhibitory, rich in collagen I and fibronectin [20].

Surrounding the fibrotic core, a distinct glial scar forms. The glial scar is composed of reactive astrocytes, neuron glial antigen 2-positive (NG2^+^) oligodendrocyte progenitor cells, and microglia in the penumbra [23]. Molecularly, reactive astrocytes respond to proinflammatory cytokines such as transforming growth factor-β (TGF-β) and fibrinogen via SMAD and signal transducers and activators of transcription signaling pathways, which drive GFAP up-regulation and hypertrophy [23]. The glial scar is marked by an abundance of CSPGs, including neurocan, versican, brevican, and NG2, whose expression peaks at 2 weeks postinjury and persists chronically [23]. Formation of the compact astrocyte scar is essentially complete by 2 to 4 weeks after injury, marking the transition to the chronic lesion stage [24].

#### Lesion borders

The glial scar establishes a spatially organized border around the fibrotic core, forming distinct proximal and distal boundaries that create a physical interface with the nonneural lesion [23]. Functionally, this border zone acts as a biochemical barrier, where inhibitory molecules such as CSPGs and tenascin C are distributed in a gradient that decreases in concentration from the core outward, potently inhibiting axonal sprouting and regeneration [5]. Paradoxically, this region also contains permissive substrates such as laminin and exhibits active angiogenesis and cellular activity, constituting a complex and contradictory microenvironment [25].

Beyond the glial border, the fibroblast-rich lesion core constitutes a distinct cellular barrier that actively impedes axonal regeneration. Forming a dense peripheral rim of collagen type I α1 chain-positive perivascular fibroblasts that abuts the astroglial scar, the fibrotic scar borders exhibit a structural organization with inflammatory macrophages localized predominantly within the inner region of the lesion core [18]. Within this fibrotic compartment, regenerating axons undergo dieback upon contact with pericyte-derived stromal cells at the glial–fibrotic interface, forming retraction bulbs that stall further extension; conversely, attenuation of this fibrotic component reduces axonal retraction and allows closer approach to the lesion margin [26].

Beyond the glial and fibrotic borders, a distinct microglial scar forms at the interface between reactive astrocytes and infiltrating immune cells. Following SCI, microglia proliferate extensively during the first week and accumulate at the lesion border, positioning themselves between the astrocytic scar on the outside and blood-derived myeloid cells on the inside [27]. Depletion of microglia disrupts astrocytic scar organization, leads to widespread inflammation and satellite lesions, and impairs locomotor recovery, indicating that the microglial border is an essential component of the neuroprotective scar that confines inflammation and supports tissue repair [27]. Mechanistically, the transmembrane receptor Plexin-B2 is up-regulated in injury-activated microglia and macrophages, where it promotes cell dispersion, contact inhibition of locomotion, and matrix compaction [28]. Genetic ablation of Plexin-B2 in myeloid cells impairs corralling, resulting in intermingling of immune cells with astrocytes, inflammatory spillover beyond the lesion core, disorganized ECM deposition, and worse motor sensory recovery [28].

#### Distant regions (rostral and caudal to the injury)

Mechanical and molecular alterations extend far beyond the primary injury site. In general, the rostral segment exhibits a more pronounced and sustained molecular and cellular response compared to the caudal segment. The rostral region shows stronger up-regulation of ECM-related genes, glial activation, Wallerian degeneration, and TGF-β2 expression, potentially leading to more pronounced secondary stiffening or ECM remodeling in this area [29]. Around lumbar motor neurons, perineuronal net (PNN) structure undergoes spatiotemporally specific “loosening followed by tightening” dynamics, affecting the plasticity of distal neural circuits [30]. Such remote effects may contribute to clinical sequelae such as chronic pain, spasticity, or compensatory plasticity.

#### Differential responses of gray and white matter

The mechanical properties of gray and white matter diverge in both the uninjured and injured spinal cord, and these differences are paralleled by distinct structural and cellular responses. White matter is consistently softer than gray matter, with gray matter being approximately twice as stiff as white matter in the uninjured state [4]. Postinjury, both undergo tissue softening, but their spatial patterns differ. Gray matter softening occurred both within and outside the visible lesion site, whereas white matter softening remained confined to the injury site [4].

Structurally, gray and white matter also follow distinct degenerative trajectories that vary by spinal level [31]. Rostral to the lesion, white matter atrophy occurs early while gray matter degeneration initially lags but subsequently accelerates, reaching similar magnitudes by 1.5 years postinjury [32]; in contrast, caudal to the injury, gray and white matter atrophy progresses concurrently from the earliest time points [32]. These spatiotemporally distinct degenerative trajectories suggest that white and gray matter are governed by fundamentally different cellular and molecular programs.

At the cellular level, this regional divergence is reflected in astrocyte reactivity. White matter lesion-remote astrocytes acquire a distinct reactive state marked by communication network factor 1 up-regulation, which governs local microglial lipid metabolism and phagocytic function to mediate white matter repair [33]. Gray matter astrocytes, by contrast, primarily engage in synaptic remodeling and circuit modulation [33]. Furthermore, white-matter-derived astrocytes migrate into the gray matter to form a distinct reactive subpopulation characterized by insulin-like growth factor binding protein 2 (Igfbp2) up-regulation, while resident gray matter astrocytes markedly diminish [34]. Thus, this injury-induced, white-matter-originating astrocyte subpopulation may represent a novel therapeutic target. Molecularly, the white matter exhibits a more rapid and robust response than the gray matter during the acute phase, consistent with this regional specialization.

## Mechanosensing Receptor Families in SCI

Following SCI, cells within the lesion and surrounding penumbra are exposed to a profoundly altered mechanical microenvironment characterized by dynamic changes in tissue stiffness, local tension, and ECM composition. To sense and adapt to these biomechanical cues, cells use a repertoire of mechanosensitive receptors that transduce physical forces into biochemical signals, critically influencing postinjury pathology and repair.

### Ion-channel-type receptors: Piezo and transient receptor potential families

**Fig. 2.**
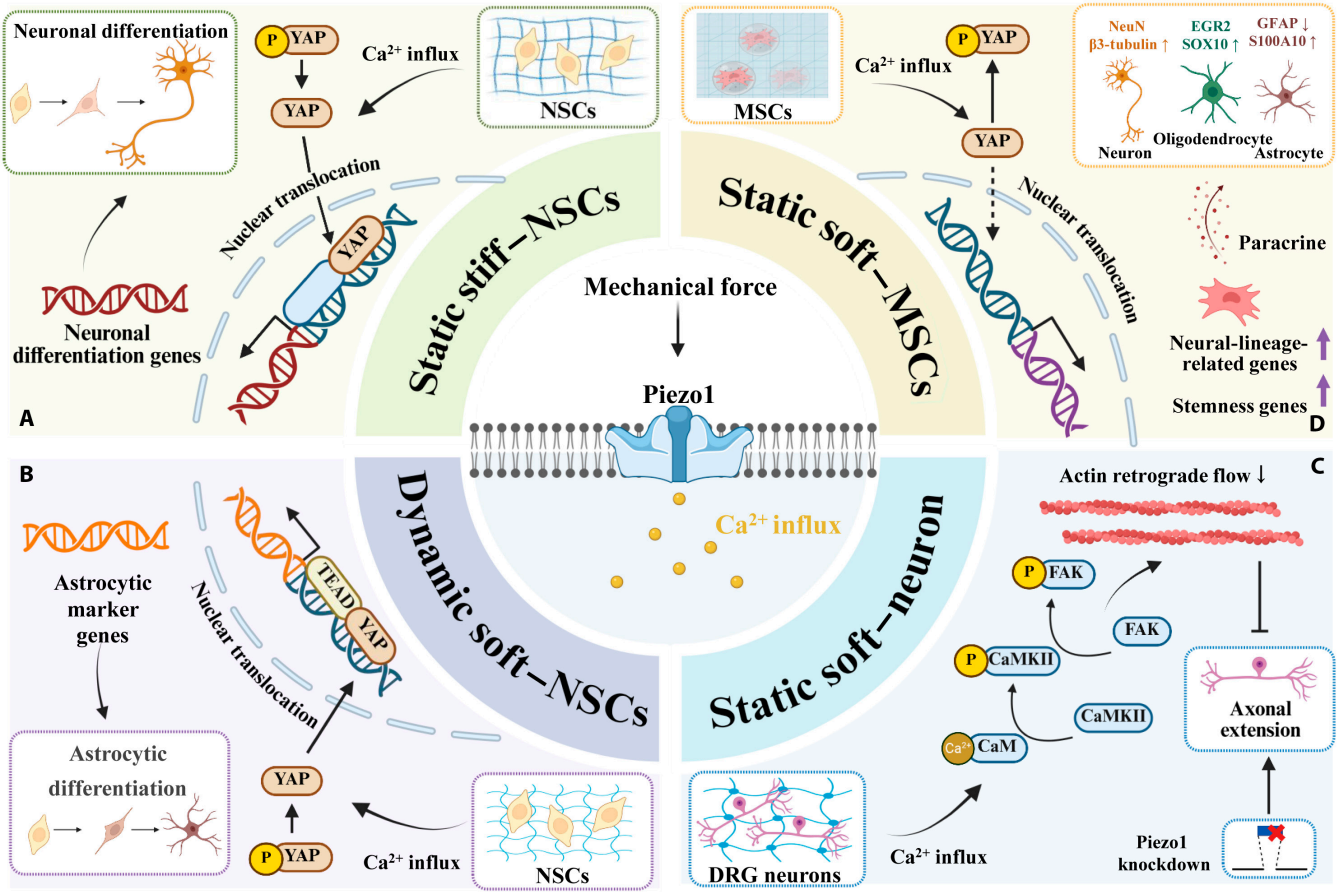
Piezo1 mediates context-dependent mechanical signal transduction after spinal cord injury. Piezo1 senses matrix stiffness, triggering Ca^2+^ influx, which leads to context-dependent outcomes following spinal cord injury. (A) In neural stem cells (NSCs) on static stiff substrates, Piezo1-mediated Ca^2+^ influx promotes yes-associated protein (YAP) dephosphorylation, nuclear translocation, and activating neuronal differentiation genes. (B) Conversely, on dynamically softening alginate hydrogels, the similar Piezo1–YAP signaling axis results in the activation of astrocytic marker genes. (C) In mesenchymal stem cells (MSCs) cultured on static soft substrates, Piezo1-mediated Ca^2+^ influx promotes YAP phosphorylation and cytoplasmic retention, which relieves repression of stemness genes, activates neural-lineage-related genes, and enhances paracrine function. (D) In dorsal root ganglion (DRG) neurons, Piezo1 activation initiates a Ca^2+^–calmodulin-dependent protein kinase II (CaMKII)–focal adhesion kinase (FAK)–actin signaling cascade that ultimately inhibits axonal extension on soft, nonpermissive substrates; conversely, Piezo1 knockdown enhances axon growth. TEAD, transcriptional enhanced associate domain; CaM, calmodulin; EGR2, early growth response 2. (Created with BioRender.com.)

The Piezo family, particularly Piezo1, emerges as a crucial mechanosensor that interprets the complex mechanical microenvironment of the injured spinal cord (Fig. 2). Functioning as a primary mechanotransducer, Piezo1 converts extracellular mechanical cues into intracellular biochemical signals by mediating Ca^2+^ influx, thereby regulating downstream effectors such as yes-associated protein (YAP) and orchestrating diverse cellular outcomes. The direction and consequence of Piezo1 activation are profoundly context dependent, governed by cell type, substrate mechanics, and the ensuing signaling cascade.

In neural stem cells (NSCs), Piezo1 couples mechanical signals to fate specification through YAP-dependent transcriptional regulation, but the resulting lineage outcome depends on both the absolute stiffness and the dynamic properties of the substrate. On static stiff substrates, myosin-II-dependent traction forces activate Piezo1 to promote YAP nuclear translocation, favoring neuronal differentiation while suppressing astrogenesis [6]. Pharmacological inhibition of Piezo1 with grammostola spatulata mechanotoxin 4 or small-interfering-RNA-mediated knockdown reduced neuronal differentiation while promoting astrogenesis and concomitantly decreased nuclear YAP localization [6]. Conversely, on dynamically softening alginate hydrogels, Piezo1-mediated Ca^2+^ influx synergizes with increased F-actin assembly and cytoskeletal tension to also drive YAP nuclear translocation, yet this results in the activation of astrocytic marker genes via YAP–transcriptional enhanced associate domain (TEAD)-mediated transcription [35]. The observation that YAP nuclear entry is required for both neuronal and astrocytic specification in NSCs indicates that lineage outcome is determined by factors beyond YAP localization alone. The absolute stiffness of the substrate differs by orders of magnitude between the 2 systems and dictates the magnitude of membrane tension and the intensity of Piezo1-mediated Ca^2+^ influx. Superimposed on this static stiffness difference, the progressive decross-linking of alginate hydrogels introduces dynamic mechanical perturbations that are absent on rigid static substrates, encoding qualitatively distinct signals that may guide YAP toward different transcriptional partners. The cell-intrinsic transcriptional landscape further shapes how these signals are interpreted, although which specific cofactors engage YAP under distinct mechanical conditions remains to be experimentally determined.

In mesenchymal stem cells (MSCs), Piezo1–YAP coupling follows a distinct logic, whereby moderate stiffness favors cytoplasmic YAP functions over nuclear transcriptional activity. On static soft substrates (~1.2 kPa), Piezo1 activation reduces nuclear YAP accumulation and promotes cytoplasmic retention, accompanied by up-regulation of neural-lineage-related genes, such as *EGR2* and *SOX10*, and enhanced paracrine secretion of neurotrophic factors [36]. This outcome illustrates another logic of Piezo1–YAP coupling, whereby moderate stiffness in a 3-dimensional (3D) architecture favors cytoplasmic YAP functions over nuclear transcriptional activity.

In mature neurons, Piezo1 governs axonal regenerative capacity through a fundamentally distinct mechanism that bypasses YAP/transcriptional coactivator with PDZ-binding motif (TAZ) entirely, instead coupling mechanical signals directly to local cytoskeletal dynamics. In dorsal root ganglion (DRG) neurons, Piezo1 is enriched in growth cones and is preferentially activated on soft substrates (0.15 to 5 kPa), where it acts as a key mediator of stiffness-dependent axon regeneration. On soft, nonpermissive substrates, Piezo1 activation initiates a Ca^2+^–calmodulin-dependent protein kinase II (CaMKII)–focal adhesion kinase (FAK)–actin signaling cascade that ultimately inhibits axonal extension, with Piezo1 knockdown enhancing axon growth both in vitro and in vivo while accelerating functional sensory recovery, thereby functioning as a molecular brake under unfavorable mechanical conditions [37]. This YAP-independent mechanism highlights a key signaling bifurcation downstream of Piezo1. In stem cells, Ca^2+^ signals converge on YAP/TAZ-mediated transcriptional regulation, whereas in postmitotic neurons, Ca^2+^ is directly coupled to local cytoskeletal dynamics at the growth cone. The spatial restriction of Piezo1-mediated Ca^2+^ transients to growth cones further enables subcellular control of axon behavior without engaging global transcriptional reprogramming. Collectively, these findings establish Piezo1 as a context-dependent mechanosensory rheostat that integrates stiffness magnitude, matrix dynamics, and cell-intrinsic programs to determine whether the biological output is survival, fate specification, or axon regeneration. Nevertheless, translating these mechanistic insights into therapeutic strategies for SCI will require direct in vivo validation in injury-specific models, as the functional contribution of Piezo1 to endogenous spinal cord repair remains to be rigorously established.

The transient receptor potential (TRP) channel family represents another critical class of mechanosensitive ion channels. Distinct TRP members contribute to mechanotransduction through diverse mechanisms and cellular contexts. Notably, TRP vanilloid 1 (TRPV1) is functionally expressed in the growth cones of DRG neurons. Interestingly, unlike Piezo1, pharmacological inhibition of TRPV1 suppresses axon regeneration across a range of substrate stiffnesses, suggesting a stiffness-independent regulatory function in axon growth [37].

### Adhesion-based receptors: Integrins and cadherins

**Fig. 3.**
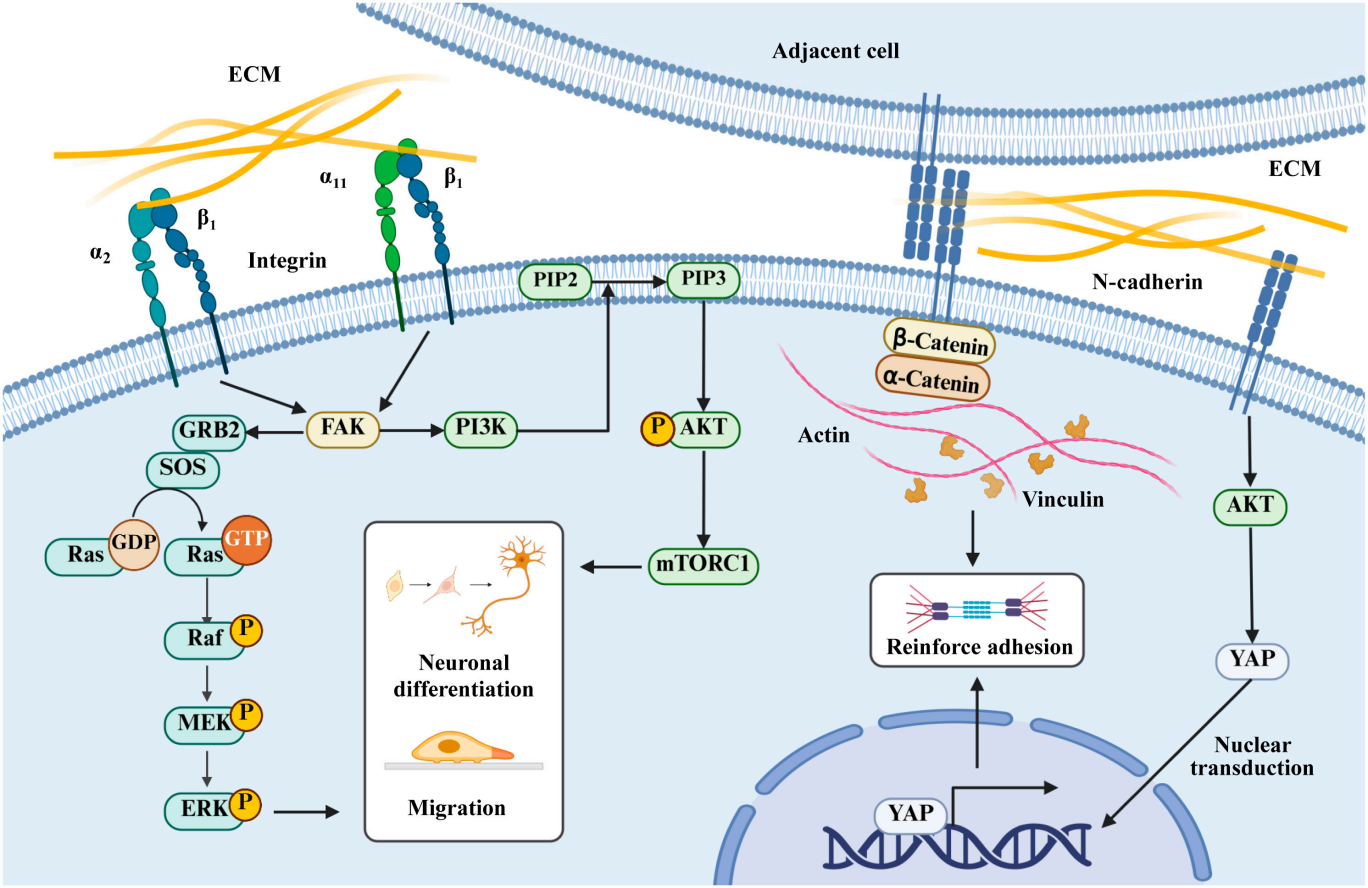
Integrins and cadherins in post-spinal-cord-injury cellular responses. Following spinal cord injury, integrins and cadherins act as key mechanosensors mediating cell–extracellular matrix (ECM) and cell–cell interactions, respectively. Integrins, exemplified by integrin β1 (ITGB1) partnered with α subunits including ITGA2 and ITGA11, bind lineage-specific ECM matrices to activate PI3K–AKT and mitogen-activated protein kinase (MAPK)–extracellular-signal-regulated kinase (ERK) pathways, promoting neural stem cell migration and neuronal differentiation. N-cadherin mediates cell–cell adhesion at adherens junctions. It connects to the actin cytoskeleton via force-sensing molecules including α-catenin and vinculin, forming a tension-responsive module that reinforces adhesion. In addition, N-cadherin can signal through the AKT/yes-associated protein (YAP) pathway to further strengthen cell–cell adhesion and stabilize junctions. PI3K, phosphatidylinositol 3-kinase; AKT/PKB, protein kinase B; mTORC1, mammalian target of rapamycin complex 1; GDP, guanosine diphosphate; GTP, guanosine triphosphate; PIP2, phosphatidylinositol 4,5-bisphosphate; PIP3, phosphatidylinositol 3,4,5-trisphosphate; GRB2, growth factor receptor-bound protein 2; SOS, son of sevenless; MEK, mitogen-activated protein kinase kinase. (Created with BioRender.com.)

Integrins represent a primary mechanism for cells to sense both biochemical and mechanical properties of the ECM (Fig. 3). Following SCI, specific integrin subunits are differentially up-regulated, among which integrin β1 (ITGB1) is consistently highlighted as a central mediator. Upon mechanical activation, ITGB1 recruits downstream effectors including integrin-linked kinase and phospho-focal adhesion kinase, initiating prosurvival and prodifferentiation signaling cascades in NSCs [7]. The intensity of integrin-mediated signaling is further modulated by matrix stiffness, which regulates integrin clustering and adhesion strength. The functional outcome of ITGB1 signaling is further diversified by its α-subunit binding partners, which tailor cellular responses to specific matrix environments. In stem-cell-based therapies, up-regulation of ITGA2 and ITGA11 (paired with ITGB1) on lineage-specific matrices activates phosphatidylinositol 3-kinase (PI3K)–AKT and mitogen-activated protein kinase (MAPK)–extracellular-signal-regulated kinase (ERK) pathways, promoting NSC migration and neuronal differentiation [38].

In addition, fibronectin–ITGB1 engagement contributes to microglial inflammation after SCI [39], although this effect is primarily driven by ligand–receptor recognition rather than force-dependent mechanotransduction. This fibronectin–integrin interaction also mediates mechanobiology-related ECM remodeling; soluble fibronectin derived from plasma is assembled into an insoluble fibrillar matrix beginning at 7 d post-SCI, a process mediated by integrin α_5_β_1_ expressed on infiltrating macrophages/microglia and fibroblasts [40]. Therapeutic targeting of integrins has been accomplished through arginine–glycine–aspartic acid (RGD) peptide-functionalized biomaterials, decellularized ECM scaffolds, and stiffness-optimized substrates that direct integrin-mediated adhesion and differentiation [41].

Classical cadherins, particularly N-cadherin (CDH2), mediate cell–cell adhesion and mechanotransduction at adherens junctions (Fig. 3). In SCI, the collagen I–ITGB1–N-cadherin signaling axis is implicated in astrocyte activation and fibrotic scar formation [42]. On the other hand, biomimetic scaffolds that up-regulate CDH2 in NSCs can promote a proneuronal niche via the AKT/YAP pathway [43]. These effects are primarily driven by biochemical signaling rather than direct force sensing. By contrast, the core mechanotransductive function of cadherins resides in their intracellular link to the actin cytoskeleton. Tension-induced unfolding of α-catenin recruits vinculin, reinforcing the adhesion and stabilizing junctions [42]. This mechanism is potentially targetable to modulate barrier integrity or glial reactivity post-SCI.

### Enzyme-linked and other signaling receptors

The protein tyrosine phosphatase receptor σ (PTPσ) is a high-affinity receptor for inhibitory CSPGs. Its expression is up-regulated in the SCI penumbra, where it colocalizes with CSPGs [44]. PTPσ activation stabilizes growth cone adhesion, leading to dystrophic arrest and inhibition of axon regeneration by suppressing ERK1/2 phosphorylation [45]. Intracellular σ peptide, a competitive peptide mimetic of the PTPσ wedge domain with a TAT sequence for cell membrane penetration, blocks PTPσ function to promote axonal regeneration and improve functional recovery, highlighting its therapeutic potential [45]. Beyond pharmacological approaches, PTPσ might also be targeted via cell-derived exosomes that modulate Rho/rho-associated coiled-coil containing protein kinase (ROCK) signaling to alleviate CSPG inhibition and facilitate axonal growth.

Although not direct mechanosensors, transcriptional coactivators YAP and TAZ are central downstream effectors in mechanotransduction, translating mechanical cues into gene expression changes. Their nucleocytoplasmic shuttling is intensely regulated by mechanical cues from the ECM and cytoskeleton [36]. In SCI, softer substrates or dynamic hydrogels often promote YAP cytoplasmic retention or specific activation patterns associated with neurogenic differentiation [36,46]. Conversely, on stiff substrates mimicking fibrotic scars, nuclear YAP/TAZ activity may drive astrocyte activation and pathological ECM remodeling [47].

The small guanosine triphosphatase ras homolog family member A (RhoA) acts as a major signaling hub that integrates diverse upstream signals, eliciting cell-type-specific outcomes following SCI. In neurons, its activation promotes actin compaction via myosin II, restraining microtubule extension to inhibit axon regeneration [48]. RhoA knockdown promotes axonal growth and reduces glial scar formation, while microtubule-stabilizing drugs represent alternative strategies to bypass RhoA-mediated growth inhibition, albeit with potential inflammatory side effects [16]. In astrocytes, RhoA controls injury-induced astrogliosis and CSPG production through myosin II by activating YAP signaling, and its ablation is antiregenerative [48]. In microglia, RhoA is essential for SCI repair by promoting glycolysis via the Rho guanosine-triphosphatase-activating protein 25/hypoxia-inducible factor-1 pathway, thereby supporting microglial proliferation, phagocytosis, and migration [49].

### Mechanosignaling convergence and cross-talk

The mechanosensitive pathways described above converge on a set of downstream effectors, creating functional intersections that allow signals from distinct receptors to be integrated or opposed before determining cellular outcomes [50,51]. YAP/TAZ represents the most prominent of these convergence points. Piezo1-mediated Ca^2+^ influx [6], N-cadherin engagement via CDH2 [43], and integrin clustering through RGD peptides [52] all modulate YAP nuclear localization, yet the biological consequence of YAP activation differs fundamentally between NSCs, where it promotes neuronal differentiation [6,43], and astrocytes, where it restricts reactive gliosis and CSPG production [48]. This indicates that the upstream receptor identity is less instructive for cell fate than the cell-type-specific transcriptional partners available to YAP upon nuclear entry, positioning YAP as a molecular integrator that translates diverse mechanical inputs into lineage-appropriate outputs.

The cytoskeleton serves as a second critical node where mechanosignaling pathways intersect. Both RhoA and Piezo1 regulate F-actin dynamics, although through distinct mechanisms and with divergent functional consequences. In neuronal growth cones, RhoA activates myosin II to compact F-actin and impede microtubule protrusion [48], while Piezo1 triggers a CaMKII–FAK cascade that slows F-actin retrograde flow [37], with the 2 pathways cooperatively gating axon extension in response to inhibitory substrates. In astrocytes, RhoA-driven actomyosin contraction and Piezo1-driven Wnt7b–Ca^2+^ signaling both modulate cytoskeletal architecture and cellular stiffness [48,53], yet their relative contributions to glial scar mechanics and whether they operate synergistically or redundantly remains unknown.

CSPG signaling illustrates a third mode of pathway integration through reciprocal regulation. The PTPσ receptor transduces CSPG signals to suppress Erk1/2 phosphorylation and stabilize growth cone arrest in neurons [45], while concurrently mediating CSPG endocytosis and clearance in neural progenitor cell (NPC)-derived astrocytes [44]. These opposing functions create a potential feedback circuit wherein the astrocytic clearance of CSPGs via PTPσ reduces the ligand available to activate neuronal PTPσ, thereby linking the ECM remodeling activity of glial cells to the growth competence of axons. Furthermore, CSPGs signal through the RhoA/ROCK axis independently of PTPσ, indicating that the inhibitory activity of CSPG-rich scars is distributed across multiple receptors and cannot be fully overcome by targeting a single CSPG receptor [48].

## Cell-Type-Specific Mechanoresponses

**Fig. 4.**
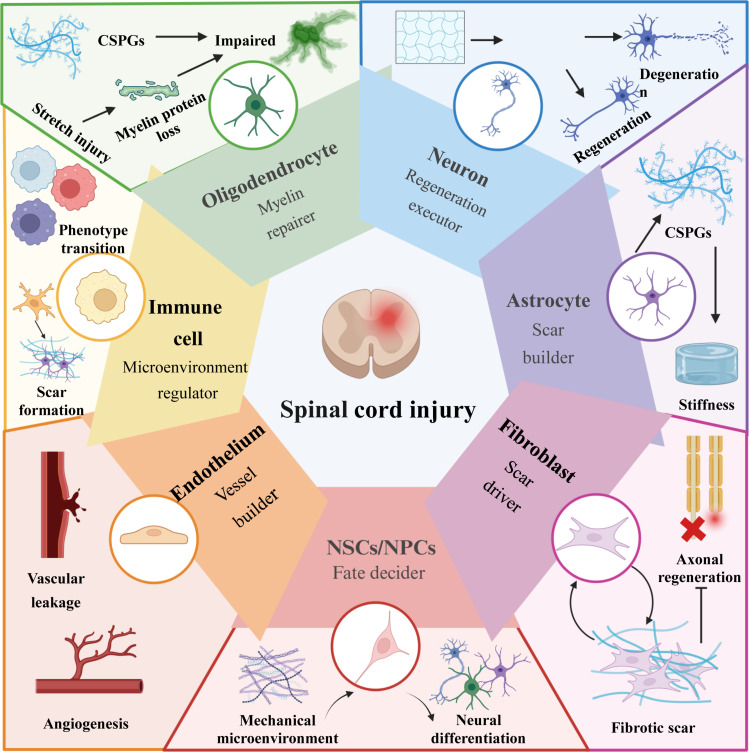
Cell-type-specific mechanoresponses in spinal cord injury. Seven major cell types involved in post-spinal cord injury (SCI) mechanobiology are grouped by their functional roles in regeneration, scar formation, or supportive processes. Each cell type exhibits distinct responses to the mechanical microenvironment, collectively determining the balance between neural repair and fibrotic scarring. NSCs/NPCs, neural stem/progenitor cells; CSPGs, chondroitin sulfate proteoglycans. (Created with BioRender.com.)

The pathological progression following SCI is fundamentally orchestrated by a mechanically driven cellular network. The initial physical disruption and subsequent dynamic remodeling of the ECM create a continuously evolving mechanical landscape. Distinct cell types, through their specific mechanosensing and response apparatus, critically dictate the balance between neural regeneration and scar formation (Fig. 4). A comprehensive overview of mechanosensitive receptors, downstream pathways, and cellular outcomes for each major cell type is provided in Table 2.

**Table 2.** Cell-type-specific mechanosensing and mechanotransduction pathways after SCI

Cell type	Mechanosensitive receptors	Downstream pathways	Mechanical response	Therapeutic implication	References
NSC/NPC	Piezo1, YAP/TAZ, integrins (ITGA2, ITGA11), N-cadherin (CDH2)	ERK/AKT, YAP nuclear shuttling, AKT/YAP	Context-dependent differentiation; aligned topography directs migration	Dynamic hydrogels, N-cadherin functionalization	[6,35,38,43,46,52]
Neuron	Piezo1, integrins, TRPV1	Ca^2+^–CaMKII–FAK–actin, Rho/ROCK	Stiffness-dependent axon growth inhibition; soft matrix promotes regeneration	Piezo1 inhibition, soft biomaterials	[37,57]
Astrocyte	Piezo1, integrin β1, N-cadherin	RhoA/ROCK, YAP/TAZ, TLR2	Stiffness-induced reactivity, CSPG deposition, scar formation	Soft hydrogels, Rho/ROCK inhibitors	[14,48,59]
OPC/oligodendrocyte	Integrins, PTPσ	RhoA/ROCK, Erk1/2	Soft matrix promotes differentiation and myelination; stiff matrix inhibits myelination	ChABC, compliant scaffolds	[57,64–66]
Microglia/macrophage	Integrins	PI3K/AKT, NF-κB, cGAS–STING, PI3K-Akt-mTOR	Stiffness/topography-guided phenotype transition	Aligned scaffolds, IL-4, anti-inflammatory hydrogels, electrospun nanofiber scaffold	[10,52,67,69,118]
Fibroblast/pericyte	Integrin β1, LRP6	Wnt/β-catenin, YAP/TAZ	Stiffness-dependent collagen deposition, fibrotic scarring	Mechanosignaling blockade, soft scaffolds	[20,74]
Endothelial cell	Integrins	PI3K/AKT	Stiffness-dependent tube formation; ECM support required for angiogenesis	Biomimetic stiffness, VEGF delivery	[10,77,78]

AKT, protein kinase B; CaMKII, calmodulin-dependent protein kinase II; CDH2, N-cadherin; cGAS, cyclic guanosine monophosphate–adenosine monophosphate synthase; CSPG, chondroitin sulfate proteoglycan; ECM, extracellular matrix; ERK, extracellular-signal-regulated kinase; FAK, focal adhesion kinase; IL-4, interleukin-4; ITGA2, integrin α2; ITGA11, integrin α11; LRP6, low-density lipoprotein receptor-related protein 6; NF-κB, nuclear factor κB; NSC, neural stem cell; NPC, neural progenitor cell; OPC, oligodendrocyte precursor cell; PI3K, phosphoinositide 3-kinase; PTPσ, protein tyrosine phosphatase receptor σ; RhoA, ras homolog family member A; ROCK, rho-associated coiled-coil containing protein kinase; STING, stimulator of interferon genes; TAZ, transcriptional coactivator with PDZ-binding motif; TLR2, Toll-like receptor 2; TRPV1, transient receptor potential vanilloid 1; VEGF, vascular endothelial growth factor; YAP, yes-associated protein; ChABC, chondroitinase ABC.

### Endogenous neural stem/progenitor cells

The recruitment and differentiation of endogenous NSCs/NPCs constitute a pivotal regenerative response that is precisely regulated by the mechanical properties of their niche. Substrate stiffness serves as a primary determinant of NSC fate, yet the reported direction of its influence varies with experimental context. In some studies using composite scaffolds rich in native ECM components, soft matrices promote neuronal differentiation [52]. Conversely, using inert polyacrylamide gels, Pathak and colleagues [6] found that stiff substrates favor neurogenesis via Piezo1–YAP signaling, while soft substrates suppress astrogenesis. Adding further complexity, dynamically softening alginate hydrogels in the presence of serum can instead enhance astrocytic differentiation through Piezo1-mediated Ca^2+^ influx and YAP activation [35]. Collectively, these findings indicate that NSC fate is governed by an interplay of absolute stiffness, matrix composition, viscoelasticity, and biochemical context, rather than by stiffness alone.

Beyond stiffness, the structural topography of the ECM, including aligned fiber architectures, provides essential contact guidance to direct NSC migration and process outgrowth [54]. Furthermore, when presented within a permissive physical framework such as a decellularized spinal cord (DSC) matrix, specific biochemical components of the ECM can potently activate proneurogenic integrin signaling pathways, including ITGA2/11–ERK/AKT, thereby further enhancing neuronal commitment [38]. Therefore, a mechanically permissive niche, characterized by optimal compliance, instructive topography, and supportive adhesive cues, is indispensable for effectively harnessing the intrinsic regenerative capacity of endogenous stem cells.

### Neurons

Neuronal regeneration is not a passive occurrence but an active process in which growth cones interpret the local mechanochemical microenvironment. Primarily, physical topographical cues provide a foundational roadmap for growth. Axons exhibit robust contact guidance, preferentially elongating and aligning along oriented nanofibers within engineered scaffolds such as aligned methacrylated gelatin (GelMA) or polycaprolactone fibers [54–56]. This directed growth constitutes a direct biomechanical response to anisotropic structural features. Concurrently, substrate stiffness acts as a critical regulator of cell fate. Soft matrices (approximately 0.1 to 1 kPa), which mimic the compliance of healthy CNS tissue, are generally permissive for neurite extension under standard culture conditions [52]. In contrast, stiffer environments, often resembling fibrotic scars, are inhibitory in the absence of countervailing topographical or biochemical cues. This sensitivity to stiffness is mediated by mechanotransduction pathways involving integrins, Piezo channels, and downstream effectors such as Rho/ROCK [57].

Furthermore, this mechanical framework is overlaid with biochemical instruction. Inhibitory molecules such as CSPGs within stiff scars potently activate Rho/ROCK signaling, leading to growth cone collapse [57]. Conversely, supportive ECM components or neurotrophic factors delivered via compliant hydrogels synergize with permissive mechanical cues to enhance neuronal survival, axonal growth, and synaptic integration [58]. Thus, neuronal regeneration represents an integrated outcome shaped by topographical guidance, matrix compliance, and embedded biochemical signals.

### Glial cells

#### Astrocytes

Astrocytes exhibit exquisite sensitivity to mechanical strain, which is directly transduced through Piezo1 to initiate reactive programs [53]. Their subsequent behavior is profoundly regulated by ECM stiffness. Stiffer substrates drive astrocytes toward a reactive, scar-forming phenotype characterized by cytoskeletal reorganization and sustained activation, accompanied by elevated vimentin and vinculin expression [59]. Critically, this establishes a self-reinforcing feedback loop: Astrocyte activation promotes local ECM stiffening, which, in turn, further exacerbates astrogliosis. Indeed, following SCI, local mature astrocytes undergo permanent transcriptional reprogramming, characterized by persistent down-regulation of homeostatic functions and sustained up-regulation of ECM organization and inflammatory response genes, which collectively establish a chronically reactive state that perpetuates the fibrotic environment [60]. Interventions using soft biomaterials or Rho/ROCK inhibitors can disrupt the loop, thereby reducing astrocyte reactivity and CSPG deposition [61]. Alternatively, activation of Toll-like receptor 2 (TLR2) on astrocytes has been shown to reduce CSPG deposition and promote functional recovery after SCI [14]. Moreover, astrocyte heterogeneity dictates distinct patterns of ECM production, with ependymal-derived subsets failing to synthesize CSPGs and instead creating a permissive bridge for axonal sprouting, in contrast to the barrier-forming phenotype of locally derived mature astrocytes [62,63].

#### Oligodendrocyte lineage cells

Myelin regeneration is similarly mechanosensitive. Oligodendrocyte precursor cells (OPCs) require a soft yet mechanically supportive microenvironment for efficient differentiation into myelinating oligodendrocytes. Using hydrogel-based micropillar arrays that mimic axonal stiffness, it has been demonstrated that while excessively soft substrates (0.5 kPa) impair myelin formation, substrates with stiffness approximating native axons (5 kPa) promote robust myelin wrapping [64]. Pathological tissue stiffening following SCI may therefore disrupt the mechanical niche required for OPC differentiation and remyelination. Moreover, inhibitory CSPGs deposited after SCI, such as versican-V1, directly impair OPC process extension and their maturation into myelinating oligodendrocytes [65], compounding the detrimental effects of an altered mechanical microenvironment on myelin repair. Furthermore, mechanical stretch injury has been shown to directly impair oligodendrocyte function by triggering intracellular Ca^2+^ release and subsequent Erk1/2 activation, leading to transient myelin protein loss without cell death [66]. This mechanotransduction mechanism may contribute to white matter pathology following traumatic CNS injury.

### Immune cells

**Fig. 5.**
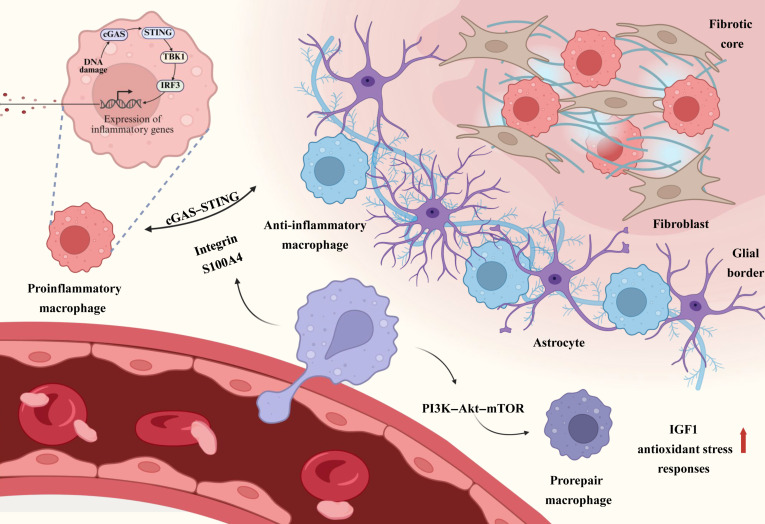
Phenotypic and spatial distribution transition of macrophages after spinal cord injury. Following spinal cord injury, loss of vascular mechanical integrity facilitates macrophage infiltration into the injury site. Phenotypic transition between proinflammatory and anti-inflammatory states is regulated by cyclic guanosine monophosphate–adenosine monophosphate synthase (cGAS)–stimulator of interferon genes (STING) and integrin-mediated signaling, as well as by S100A4 released from neural progenitor cells. Functionalized biomaterial scaffolds further promote a prorepair transcriptional state via phosphoinositide 3-kinase (PI3K)–Akt–mammalian target of rapamycin (mTOR) pathway activation, characterized by insulin-like growth factor 1 (IGF1) up-regulation and enhanced antioxidant stress responses. Spatially, proinflammatory macrophages predominate within the fibrotic core, sustaining inflammatory pathology. Anti-inflammatory are enriched at the glial scar border, where they contribute to wound healing and extracellular matrix remodeling. TBK1, TANK-binding kinase; IRF3. interferon regulatory factor 3. (Created with BioRender.com.)

Immune cells act as dynamic sensors and remodelers at the injury site, with their phenotype and function being highly responsive to mechanical cues. Their initial infiltration is facilitated by the loss of vascular mechanical integrity, which increases permeability [10]. Biomaterial scaffolds with defined stiffness, porosity, and biochemical signals can actively direct proinflammatory macrophages toward anti-inflammatory phenotypes through pathways such as cyclic guanosine monophosphate–adenosine monophosphate synthase (cGAS)–stimulator of interferon genes (STING) and integrin-mediated signaling, as well as through regulation by S100A4 released from NPCs [52]. Beyond this classical polarization, functionalized biomaterial scaffolds have been shown to promote the transition of microglia/macrophages toward broader reparative transcriptional states via PI3K–Akt–mammalian target of rapamycin (mTOR) pathway activation, characterized by insulin-like growth factor 1 (IGF1) up-regulation and enhanced antioxidant stress responses, thereby remodeling the post-SCI immune microenvironment [67]. This functional diversity extends beyond polarization states to their roles in fibrosis and wound healing. C–X3–C motif chemokine receptor 1 (Cx3Cr1)-low expression (Cx3Cr1^lo^) macrophages populating the fibrotic core, while Cx3Cr1-high expression (Cx3Cr1^hi^) macrophages reside in the glial scar region, whereas foamy macrophages contribute to proinflammatory pathology and can be therapeutically targeted via CD36 inhibition (Fig. 5) [68].

Compared with macrophages, microglia exhibit even greater heterogeneity after SCI. Single-cell transcriptomic studies have instead revealed at least 8 distinct microglial subpopulations that undergo dynamic temporal transitions after injury, including a second wave of activation at day 14 that coincides with secondary neuronal loss [9]. Beyond this compositional heterogeneity, recent studies have also uncovered key metabolic and mechanical programs that underpin microglial function. Specifically, microglial RhoA signaling promotes glycolysis via the Arhgap25/HIF-1α pathway, which is essential for supporting phagocytosis and migration [49]. Meanwhile, Fascin-1 limits myosin activity to facilitate microglial migration and control ECM remodeling, thereby regulating tissue stiffness after SCI [69,70]. What’s more, activated microglia promote astrocyte proliferation and scar formation through the release of IGF1, a process that helps establish the structural border confining inflammation [27]. However, their depletion disrupts the recruitment and proper spatial containment of monocyte-derived macrophages and prevents the up-regulation of key-repair-associated genes in multiple cell types, effects that can be partially rescued by restoring microglia-dependent C–C motif chemokine ligand 2 and TLR2 signaling [71]. Notably, neonatal microglia promote scar-free healing by transiently secreting fibronectin to form ECM bridges and expressing proteinase inhibitors that rapidly resolve inflammation, properties absent in adult microglia [72].

### Stromal and endothelial cells

#### Pericytes/fibroblasts

A defining mechanoresponse in neural repair is the activation and differentiation of platelet-derived growth factor receptor β-positive stromal cells, including pericytes and fibroblasts. Following injury, these cells detach from the vasculature, migrate into the lesion core, and deposit a dense, aligned network of fibrillar ECM components such as collagen I and fibronectin [20]. This deposition process serves as a primary driver of pathological tissue stiffening. The resulting fibrotic scar constitutes a substantial physical barrier to axonal regrowth and tissue repair [73]. Importantly, the progression of fibrosis is modulated by mechanical cues from the surrounding healing milieu; for example, the stiffness of an implanted biomaterial can direct fibroblast alignment and subsequent ECM organization [74]. Beyond external mechanical cues, the mechanical behavior of these scar-forming fibroblasts is critically regulated by their cytoskeletal dynamics. Polarized microtubule networks, marked by leading-edge enrichment of detyrosinated tubulin, enable fibroblast directional migration and aligned ECM deposition, which generates long-range biomechanical constraints on regenerating axons [75]. Microtubule stabilization with low-dose taxol disrupts TGF-β signaling by preventing Smad2/3 nuclear translocation, thereby reducing fibroblast migration, fibronectin deposition, and CSPG accumulation at the lesion site, further alleviating these biomechanical constraints [76]. In chronic phases, elevated tissue stiffness further stimulates fibroblast activation, establishing a self-reinforcing cycle that perpetuates fibrotic scarring.

#### Endothelial cells

Endothelial cell responses after injury proceed through 2 distinct phases. Initially, their junctional integrity is compromised as mechanical stability is lost, resulting in vascular barrier breakdown [10]. In the subsequent reparative phase, angiogenic sprouting depends strongly on the 3D physical support and mechanical compliance provided by implanted hydrogels [77]. Furthermore, tissue-specific endothelial cells, including those from spinal cord microvasculature, exhibit optimal growth and tube formation within a specific stiffness range that resembles their native microenvironment. This mechanosensitive process is partly regulated by the PI3K–AKT signaling pathway [78]. Effective angiogenesis, supported by appropriate mechanical scaffolding and necessary growth factors, is essential to restore nutrient and waste exchange and to establish the metabolic foundation required for neural regeneration.

### Intercellular communication networks in scar formation and regeneration

The diverse cell types recruited to the SCI lesion do not act autonomously but engage in reciprocal signaling that collectively determines whether the injury environment supports or impedes repair. A key mode of intercellular communication involves the paracrine regulation of ECM composition by one cell type to control the behavior of another. Reactive astrocytes secrete fibronectin that acts on microglial ITGB1 to maintain M1 polarization and chronic inflammation within the glial scar [39], establishing an astrocyte-to-microglia instructive axis that perpetuates neuroinflammation. Conversely, NPCs release S100A4 upon neurogenic differentiation, which polarizes macrophages and microglia toward an M2 anti-inflammatory phenotype [52]. This reciprocal capacity of different glial and progenitor populations to either exacerbate or resolve inflammation through ECM protein and cytokine secretion highlights that the net inflammatory state of the lesion is the product of competing paracrine signals originating from multiple cellular sources.

A second mode of communication involves mechanical coupling between cell types through the ECM. Type A pericytes detach from the vasculature after injury and differentiate into scar-forming fibroblasts that deposit fibronectin and collagen I [20], directly increasing tissue stiffness. This stiffening, in turn, feeds back on resident astrocytes to promote YAP nuclear localization and reactive gliosis [47], creating a pericyte–fibroblast–astrocyte mechanical feedback loop that drives scar maturation. Microglia independently modulate tissue mechanics through Fascin-1-dependent regulation of myosin activity and cell migration, promoting the tissue softening that characterizes the acute to subacute injury phase [69]. The net mechanical properties of the lesion are thus not passively determined by ECM deposition alone but actively negotiated through the counterbalancing activities of softening and stiffening processes.

The organization of scar tissue into discrete compartments is governed by heterotypic cell–cell contacts. At the interface between the fibrotic core and the surrounding glial scar, pericyte-derived fibroblasts and reactive astrocytes establish aligned cell–cell contacts that define a sharp lesion border [20,74]. When pericyte-derived fibroblasts fail to detach from the vasculature, as observed in striatal ischemic lesions, this organized border does not form, and the fibrotic and glial compartments remain intermingled [20]. This observation implies that the physical segregation of the fibrotic and glial scars depends on contact-dependent communication between these 2 cell populations. Moreover, within the fibrotic compartment, fibroblasts can be directed by appropriate mechanical cues to adopt a polarized, aligned migration pattern and remodel disorganized ECM into a parallel-oriented matrix that directly supports axon extension and synapse formation [74], transforming the scar from a passive barrier into an instructive substrate through mechanical reprogramming of a specific fibroblast subset.

## Mechanical Intervention Strategies and Underlying Mechanisms

**Fig. 6.**
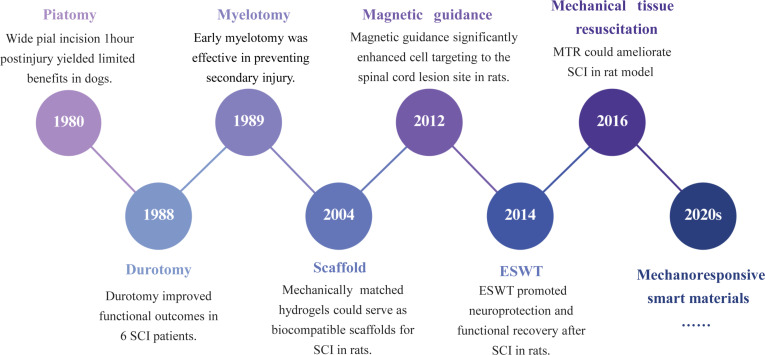
Timeline of mechanical intervention strategies after spinal cord injury. This timeline charts the emergence of key mechanical interventions over time, illustrating a historical shift from passive structural decompression to active biomechanical modulation approaches for neural repair. ESWT, extracorporeal shock wave therapy; MTR, mechanical tissue resuscitation. (Created with BioRender.com.)

Emerging interventions are shifting from passive structural decompression to active biomechanical modulation aimed at remodeling the pathological mechanical microenvironment and harnessing mechanotransduction for neural repair (Fig. 6). Key clinical trials investigating these mechanical strategies are summarized in Table 3 and Table S1.

**Table 3.** Key clinical trials of mechanical interventions for spinal cord injury

Category	Study ID^a^	Design	Current status	Population	Intervention	Key findings
**Surgical decompression**	NCT01485458	Multicenter RCT	Completed	Cervical SCI, AIS C, with stenosis (*N* = 70)	Early (<24 h) versus delayed (>2 wk)	No difference in 1-y motor score; early surgical decompression gained significantly faster recovery in first 6 mo
	NCT04034108	Single arm	Completed	SCI (AIS A) (*N* = 320)	Surgical intervention + weight-supported ambulation training	AIS conversion in 47.2% of patients
	NCT04936620	Phase III RCT (ongoing)	Recruiting	Severe acute cervical SCI, AIS A–C (*N* = 222, planned)	Duroplasty	Pending
	NCT01674764	Cohort	Completed	Acute SCI, AIS A–D (*N* = 226)	Early (≤12 h) versus delayed (>12 h)	No significant difference in QoL outcomes
	NCT05653206	Prospective, multicenter observational case series	Recruiting	Adults with acute traumatic cervical SCI, AIS D or sensory-only, without spinal instability (*N* = 50, planned)	Nonsurgical or surgical management	Pending
**Extracorporeal shock wave therapy**	NCT04474106	Phase 2/3 RCT (ongoing)	Recruiting	Acute traumatic SCI, AIS A–D, <48 h postinjury (*N* = 246, planned)	Single-session focused ESWT versus dummy head	Pending
	Comino-Suárez et al. [119]	Case report + literature review	Completed	18 M, chronic C5 AIS C, with right triceps surae spasticity (MAS 3)	rESWT + conventional rehab	Improved ankle ROM (−15°→10°), reduced muscle resistance (33%–55%) and spasticity (MAS 3→2)
	Li et al. [120]	Case report	Completed	30 F, chronic C8 AIS D, with NHO of left hip	rESWT + conventional PT	Reduced NHO size, improved hip PROM, VAS 8→0, ALP 184→86 IU/l
	Jeon et al. [121]	Case report	Completed	55 M, chronic C4 AIS A, with painful NHO of right hip	Focused ESWT	VAS 7-8→3, sitting tolerance 1 h→10 h. No significant change in NHO size
	Kang et al. [122]	Case report	Completed	51 M, L1 SCI with right heel pressure ulcer	rESWT postdebridement	Complete wound healing at 3 mo
	NCT02203994	Interventional (single-arm or RCT design unclear)	Completed	Adults with incomplete SCI and lower limb spasticity (*N* = 20)	Single-session ESWT to spastic lower limb muscles	Results not yet published
**Scaffolds**	NCT03762655	Multicenter RCT	Terminated	Acute, complete thoracic SCI (AIS A) (*N* = 20)	NSS implant	Did not meet primary end point (20% versus 30% in control)
	NCT02510365	Phase 1, single arm	Completed	Acute, complete thoracic SCI (AIS A) (*N* = 7)	NeuroRegen scaffold + BMMCs	No motor recovery; partial sensory/autonomic improvements noted

AIS, American Spinal Injury Association impairment scale; ALP, alkaline phosphatase; BMMCs, bone marrow mononuclear cells; ESWT, extracorporeal shock wave therapy; MAS, modified Ashworth scale; MSCs, mesenchymal stromal/stem cells; NHO, neurogenic heterotopic ossification; NSS, neurospinal scaffold; PROM, passive range of motion; PT, physical therapy; QoL, quality of life; RCT, randomized controlled trial; rESWT, radial extracorporeal shock wave therapy; ROM, range of motion; SCI, spinal cord injury; VAS, visual analog scale.

^a^ Trials were retrieved from ClinicalTrials.gov on 2026 April 14 by searching for “spinal cord injury” as the condition/disease, combined with intervention-related terms including “decompression”, “shock wave”, “magnetic guidance”, “scaffold”, and “biomaterial”. Additional keyword searches for “mechanical”, “biomechanical”, and “stiffness” were performed to identify mechanically relevant studies. Only studies with therapeutic intent, direct relevance to mechanical or mechanobiology-based strategies for SCI, and completed, recruiting, or active status were included. PubMed was additionally searched on the same date for published clinical studies using similar terms.

### Surgical decompression

Early surgical decompression, particularly procedures such as durotomy or pialotomy involving a full-thickness incision of the meninges performed within 24 h after injury, functions as a foundational mechanical intervention. A pooled analysis of individual patient data from 4 prospective cohorts demonstrated that surgical decompression within 24 h of acute SCI significantly improves 1-year neurological recovery, with the first 24 to 36 h representing the critical therapeutic window for optimal outcomes [79]. However, a multicenter randomized controlled trial (RCT) (NCT01485458) found that while ultraearly decompression (within 24 h) did not improve 1-year motor scores compared to delayed surgery, it significantly accelerated recovery in the first 6 months [80], possibly due to the modest cohort size and the enrollment restricted to patients with cervical stenosis without fracture or dislocation.

Surgical decompression reduces intramedullary pressure (IMP), which, in turn, restores spinal cord perfusion pressure by relieving the compressive occlusion of the microvasculature, thereby restoring blood flow to the ischemic penumbra. This mechanical relief limited progressive edema formation and cystic cavitation, suppressed secondary inflammatory infiltration and fibrotic deposition, and accelerated the recovery of the native elastic stiffness of the injured spinal cord tissue [81]. These mechanobiological improvements, particularly the reduction in IMP and the restoration of tissue elastic stiffness, are transduced into prosurvival cellular signals. Specifically, this reduction restores impaired autophagic flux, as evidenced by an increased red/yellow dot ratio of mRFP–GFP–LC3 and decreased sequestosome 1/p62 accumulation [82]. Consequently, autophagic degradation is promoted, the B cell lymphoma-2 (Bcl-2)/Bcl-2-associated X protein ratio is up-regulated to inhibit apoptosis, myelin basic protein (MBP) expression and white matter integrity are preserved, and neuronal-nuclei-positive neuronal survival is increased [82]. Thus, surgical decompression represents an optimization of an already guideline-endorsed standard, with ongoing debate centered on the incremental value of ultraearly intervention within a 24-h window rather than on the principle of decompression itself.

### Extracorporeal shock wave therapy (ESWT)

As a noninvasive biomechanical intervention, extracorporeal shock wave therapy (ESWT) delivers controlled mechanical waves to the injured tissue. These waves exert direct mechanical forces that activate mechanosensitive ion channels such as Piezo, alongside generating cavitation effects that produce microstreaming and shear stresses at the cellular level [83]. These mechanical cues are rapidly converted into intracellular biochemical signals through the engagement of downstream pathways including ERK, AKT, and PI3K/AKT [83]. Building upon this mechanotransductive foundation, low-energy ESWT promotes brain-derived neurotrophic factor (BDNF) expression in neurons, astrocytes, and oligodendrocytes via the PERK/ATF4 signaling pathway, thereby reducing demyelination, oligodendrocyte loss, and axonal damage, while improving locomotor and sensory recovery after SCI [84]. Clinically, an ongoing phase 2/3 randomized sham-controlled trial (NCT04474106) is evaluating a single session of focused ESWT delivered within 48 h of acute SCI, with change in total motor and sensory score at 6 months as the primary end point [85]. ESWT is thus positioned as a potential adjunct to standard surgical decompression and rehabilitation, with its clinical role contingent upon the results of ongoing sham-controlled trials.

### Magnetic guidance

At the cellular level, innovative strategies harness magnetic forces to remotely control cell behavior and guide neural regeneration. Superparamagnetic iron oxide nanoparticles grafted onto aligned electrospun fibers act as magnetic actuators, converting alternating magnetic field energy into cyclic mechanical forces that activate mechanosensitive ion channels and promote cytoskeletal remodeling, thereby enhancing neurite outgrowth [86]. In a related approach, under static magnetic field guidance, NSCs internalizing meso-2,3-dimercaptosuccinic acid-coated Fe_3_O_4_ nanoparticles differentiate preferentially into functional neurons via activation of the PI3K/AKT/mTOR pathway [87]. Thus, magnetic actuation promotes neuroregeneration through either extracellular mechanical stimulation or intracellular signaling pathway activation.

Building upon these principles, subsequent strategies have integrated magnetic guidance with additional therapeutic features. An Fe_3_O_4_ nanoparticle-loaded hydrogel under external magnetic stimulation reduces CSPG deposition and modulates the inflammatory microenvironment to promote neurite outgrowth [88]. Taking this concept further, an anisotropic Fe_3_S_4_ ferrofluid hydrogel orients its internal particles under a magnetic field to form aligned microstructures, directing axonal regeneration while simultaneously releasing hydrogen sulfide to exert anti-inflammatory effects via the nuclear factor κB (NF-κB) pathway [89]. Collectively, these studies illustrate a progression from isolated magnetic actuation to multifunctional platforms that combine topographical guidance, biochemical signaling, and immunomodulation for enhanced spinal cord repair. Notably, magnetic guidance strategies remain at the preclinical proof-of-concept stage and have not yet entered human trials; their eventual clinical role will likely be as a component of combinatorial biomaterial or cell-based platforms rather than as standalone therapies.

## Application of Biomaterials and Tissue Engineering in Regulating Microenvironments

Following SCI, the local mechanical milieu undergoes profound disruption characterized by tissue softening, cavitation, and the formation of mechanically disorganized inhibitory scars. Biomaterials and tissue engineering strategies are designed to counteract these pathological changes by actively constructing or modulating the physical microenvironment to support regeneration (Table 4).

**Table 4.** Strategic overview of biomaterial design strategies for spinal cord injury repair

Material strategy	Examples	Fabrication/integration	Advantages	Key response mode	Future directions
**Mechanically matched materials**	Alginate ACH [47]; HyA/Coll-IV-Fn [90], HADA/HRR [74]; PCFS [43]	Ionic-covalent dual cross-linking, freeze-drying, oxidative coupling, enzyme-catalyzed Schiff base	Minimizes host inflammation, promotes neuronal differentiation, remodels fibrotic scar, softest not always optimal	Stiffness-dependent YAP signaling, reciprocal host tissue softening with stiff implants	Dynamic stiffness modulation, integrate degradable cross-linkers for programmed softening
**Topography-guided materials**	A-DSCF [25], AFGN [91], coaxial 3D printed scaffold [42], PCFS [43]	Electrospinning with aligned collection, affinity-oriented protein immobilization, coaxial 3D printing, wet spinning with surface porogen	Directs oriented axonal growth, synergizes with ECM composition, enables phase-separated temporal functionality	Contact guidance coupled to CDH2/N-cadherin mechanotransduction	Gradient topography, integrate piezoelectric materials, patient-specific architectures
**Stress–relaxation/viscoelastic materials**	DHA-Col [92], AHA-MA/Col [93], GHP bioink [46], Zn^2+^–bisphosphonate [95], CD/HRI [96]	Static covalent network plus dynamic imine/coordination/π–π bonds, microfluidic coaxial spinning, 3D bioprinting	Independent tuning of viscoelasticity and stiffness, faster relaxation promotes neurogenesis, simultaneously enables injectability and self-healing	Motor-clutch model: Stress dissipation prolongs integrin engagement, Piezo1–YAP axis	Viscoelasticity gradients, combine with conductive components and topographical cues
**Conductive/piezoelectric materials**	CNT/GelMA [98], MoS_2_/GO/PVA [100], KNN/DSCM/GelMA [101], PMEAC [99]	Rotary electrospinning for CNT alignment, freeze-thaw physical cross-linking, UV polymerization with embedded nanoparticles, polydopamine coating	Restores tissue conductivity, wireless ultrasound-driven stimulation, multifunctional integration (conductive, ROS-scavenging, antibacterial)	Voltage-gated Ca^2+^ channels, CaMKIIb-PGC-1α mitochondrial bioenergetics pathway	Biodegradable conductive polymers, self-powered triboelectric systems, multistimuli-responsive platforms
**Immunomodulatory materials**	BWPU-dECM [103], HA–astrocyte ECM [102], Alg–Zn/SF [95], FC/FI–Cur [97]	Freeze-drying, Diels–Alder click chemistry, Zn^2+^ dynamic coordination, π–π stacking self-assembly	M2 macrophage polarization via ECM components, metal ions, or natural agents, multiomics identifies PI3K–AKT as core pathway	A20/NF-κB (Zn^2+^), ECM–integrin–FAK, structural integrity prevents inflammation	Minimal active ECM combinations, on-demand anti-inflammatory release, combined immunocellular therapy
**Cell delivery materials**	SHIELD [41], Salmon fibrin/HA/laminin [104], AFGN [91], GHP [46]	Protein–peptide heterodimerization with thermal responsiveness, enzyme-catalyzed IPN formation, affinity-oriented protein immobilization, 3D bioprinting	Shear thinning protects cells during injection, cell-type-specific mechanical optimization, instructive biochemical cues enhance delivered cell function	Temperature-responsive gelation, integrin–RGD survival signaling, dynamic mechanics plus adhesion peptides	Prevascularized organoids, integrated immunomodulation, spatially organized multicell constructs
**Smart responsive/controlled release**	Core–shell coaxial print [42], DSC/HepMA-bFGF [38], KNN/DSCM/GelMA [101], FC/FI–Cur [97]	Coaxial 3D printing for phase separation, electrostatic heparin-mediated loading, ultrasound-responsive piezoelectric particles, passive drug entrapment	Architectural encoding for programmed sequential release, external stimuli enable on-demand delivery, sustained release matches therapeutic windows	Thermo-responsive shell liquefaction, ultrasound-driven piezoelectric output, diffusion controlled by network density	Multistimuli systems, closed-loop feedback control, synergy with rehabilitation training

3D, 3-dimensional; ACH, anisotropic capillary hydrogel; A-CF, aligned collagen fibers; A-DSCF, aligned decellularized spinal cord matrix electrospun fiber; AFGN, aligned fibrin nanofiber hydrogel modified with N-cadherin–Fc; AHA, aldehyde-functionalized hyaluronic acid; AKT, protein kinase B; ALDH1L1, aldehyde dehydrogenase 1 family member L1; BDNF, brain-derived neurotrophic factor; bFGF, basic fibroblast growth factor; BP, bisphosphonate; BWPU, biodegradable waterborne polyurethane; CaMKIIb, calcium/calmodulin-dependent protein kinase IIβ; CD, DOPA-grafted chitosan; CDH2, cadherin-2; CNF, cellulose nanofiber; CNT, carbon nanotube; Coll-IV, collagen type IV; CSPG, chondroitin sulfate proteoglycan; DHA, dual-functionalized hyaluronic acid; dECM, decellularized extracellular matrix; DOPA, 3,4-dihydroxyphenylalanine; DRG, dorsal root ganglion; DSC, decellularized spinal cord; DSCM, decellularized spinal cord matrix; ECM, extracellular matrix; EDC, 1-ethyl-3-(3-dimethylaminopropyl)carbodiimide; ERK, extracellular-signal-regulated kinase; FAK, focal adhesion kinase; Fbn, fibronectin; FC, Fmoc-grafted chitosan; FI, Fmoc–peptide IKVAV; Fmoc, fluorenylmethyloxycarbonyl; Fn, fibronectin; GelMA, gelatin methacryloyl; GFAP, glial fibrillary acidic protein; GHP, GelMA/OHA hydrogel with HAVDI and RGI peptides; GO, graphene oxide; HA, hyaluronic acid; HADA, hyaluronic acid–dopamine conjugate; HA-SH, thiolated hyaluronic acid; HDI, hexamethylene diisocyanate; HepMA, heparin methacrylate; HRI, HGF–RADA16–IKAV peptide; HyA, hyaluronic acid; IL-6, interleukin-6; IL-10, interleukin-10; IPN, interpenetrating network; iPSC, induced pluripotent stem cell; KNN, potassium sodium niobate; Lam, laminin; MAPK, mitogen-activated protein kinase; MBP, myelin basic protein; MMP, matrix metalloproteinase; MoS_2_, molybdenum disulfide; MWCNT, multiwalled carbon nanotube; N-Cad, N-cadherin; NF-κB, nuclear factor κB; NHS, *N*-hydroxysuccinimide; NSC, neural stem cell; NT-3, neurotrophin-3; OHA, oxidized hyaluronic acid; OPC, oligodendrocyte precursor cell; PCFS, porous collagen fiber scaffold; PDA, polydopamine; PEG, polyethylene glycol; PEGDA, polyethylene glycol diacrylate; PGC-1α, peroxisome-proliferator-activated receptor γ coactivator 1α; PI3K, phosphoinositide 3-kinase; PLO, poly-l-ornithine; PMEAC, polycitrate-maleate-ε-polylysine/polydopamine-coated MWCNT nanocomposite hydrogel; PNIPAM, poly(*N*-isopropylacrylamide); PVA, polyvinyl alcohol; RGD, arginine–glycine–aspartic acid; RGI, BDNF mimetic peptide; RhoA, ras homolog family member A; ROS, reactive oxygen species; SCI, spinal cord injury; SF, silk fibroin; SHIELD, shear-thinning hydrogel for injectable encapsulation and long-term delivery; TEAD, transcriptional enhanced associate domain; YAP, yes-associated protein; UV, ultraviolet; HRR, HGF–(RADA)_4_–DGDRGDS.

### Biophysical cue-driven materials

#### Mechanically matched materials

The relationship between matrix stiffness and neural cell behavior depends critically on the stiffness range and biological context. Inert polyacrylamide gels and dynamically softening alginate hydrogels have established that NSC fate is governed by an interplay of absolute stiffness, matrix composition, and viscoelasticity rather than by stiffness alone, with the Piezo1 Ca^2+^ channel serving as a key mechanosensor whose functional output shifts depending on the mechanical context [6,35].

Alginate anisotropic capillary hydrogels with 4 stiffness grades (~1 to 9 kPa, tuned by polymer and crosslinker concentration) and uniform capillary architecture (~120 μm in diameter) were compared in a rat cervical hemisection model [47]. All scaffolds shared an identical poly-l-ornithine/laminin coating, isolating stiffness as the sole variable. The softest formulation (~1 kPa) minimized foreign body reaction and maximized cell infiltration and axonal regrowth, while a moderately soft formulation (~1.5 to 3 kPa) emerged as optimal by retaining linear guidance without exaggerated inflammation. Notably, the stiffest scaffolds induced measurable softening of adjacent host spinal cord tissue [47]. Hyaluronic acid (HyA) macroporous scaffolds with stiffness tuned by polymer concentration (0.90 to 3.65 kPa) were functionalized with collagen IV and fibronectin via carbodiimide cross-linking, combining mechanical matching with synergistic integrin-mediated signaling [90]. The softest formulation (<1 kPa; matching spinal cord at 0.5 to 1.2 kPa) simultaneously promoted robust DRG neurite outgrowth and a neurotrophic astrocyte phenotype. Both systems converged on ~1 kPa as optimal for minimizing inflammation, yet each revealed that very soft scaffolds risk structural instability, underscoring the need to cooptimize stiffness with architectural design [47,90].

Expanding the functional scope of mechanical matching beyond immune and neuronal modulation, an HyA–dopamine conjugate (HADA) hydrogel with stiffness graded by catechol cross-linking density (~50, ~100, and ~400 Pa) was combined with a self-assembling HGF–(RADA)_4_–DGDRGDS peptide [74]. The formulation matching spinal cord (~100 Pa) selectively enriched fibroblasts that transformed disorganized fibrotic scar into ordered, proregenerative ECM. Dual-network porous collagen fibers (PCFS) fabricated by enzyme-catalyzed Schiff base cross-linking and wet spinning exhibited a Young's modulus of ~2.42 kPa and independently corroborated that matching native spinal cord mechanics promotes neuronal differentiation [43]. Together, these studies demonstrate that optimal stiffness for neural regeneration is context dependent (Table 5).

**Table 5.** Optimal stiffness ranges for neural repair summarized by cell type and material context

Cell type	Material type	Optimal stiffness	References
Human neural stem cells (neurogenesis)	Qgel-based substrates	750 kPa	[6]
	Porous collagen fibers	2.42 kPa	[43]
Human neural stem cells (astrogenesis)	Alginate hydrogel	~1 kPa	[35]
Astrocyte	Hyaluronic acid scaffold	0.9 kPa	[90]
DRG neuron	Hyaluronic acid scaffold	0.9 kPa	[90]
	Porous collagen fibers	2.42 kPa	[43]
Fibroblast	Hyaluronic acid–graft–dopamine	0.1 kPa	[74]

DRG, dorsal root ganglion.

Mechanically matched materials have converged on an optimal stiffness window, yet persistent challenges remain. Very soft formulations that best approximate spinal cord mechanics often suffer from structural instability in vivo and nonlinear axonal regrowth within the scaffold, limiting their capacity to provide sustained guidance during the chronic phase of regeneration [47]. Furthermore, stiffness matching alone does not guarantee functional integration, as the dynamic mechanical environment of the injury site evolves over time, whereas most current materials present static mechanical properties.

#### Topography-guided materials

Beyond isotropic stiffness, imparting anisotropic topography provides essential contact guidance that mimics the longitudinal organization of spinal tracts. Aligned DSC matrix electrospun fibers (A-DSCFs) exemplify the synergy of structural and compositional biomimicry, as the aligned nanofiber architecture combined with 83 retained ECM proteins outperformed aligned collagen fibers in promoting OPC differentiation, myelination, and axonal extension. Functional dissection identified laminin and agrin as specific drivers of myelination, while collagen IV, agrin, and laminin synergistically enhanced axon growth and reduced cystic cavity formation partly through M2 macrophage polarization [25].

The integration of topography with cell adhesion signals has been further refined using aligned fibrin nanofiber hydrogel modified with N-cadherin–Fc (AFGNs) [91]. The oriented immobilization of N-cadherin–Fc via protein A affinity capture preserves full bioactivity of the extracellular domain. When combined with NSC transplantation in a complete transection model, AFGN promoted long-term cell retention, mature neuronal differentiation, robust myelination, synapse formation, and motor function recovery [91]. Similarly, aligned collagen fibers with porous surface protrusions promote neuronal differentiation of NSCs via CDH2–AKT/YAP signaling, demonstrating that specific adhesion receptors couple topographical cues to mechanotransduction pathways [43].

Architectural control can also be achieved through advanced manufacturing. A coaxial 3D printed scaffold with a gelatin/cellulose nanofiber shell and a HyA dual-network hydrogel core exemplifies phase-separated functionality [42]. The shell rapidly degrades at 37 °C to release the reactive oxygen species (ROS) scavenger metalloporphyrin Mn(III) tetrakis(4-benzoic acid) porphyrin, shielding endogenous NSCs from acute oxidative stress, while the core remains stable for over 60 d, providing sustained N-cadherin-mediated guidance for NSC migration and neuronal differentiation [42]. The parallel linear architecture of the printed construct further contributes to directional axonal regrowth.

A principal limitation of topography-guided materials is the reliance on anisotropic architectures that, while effective for linear axon guidance, do not recapitulate the complex multidirectional circuitry of the native spinal cord. In addition, the long-term stability of aligned structures in the inflammatory lesion microenvironment remains uncertain, as enzymatic degradation may progressively erase topographical cues. Scalable manufacturing of precisely aligned constructs with clinically relevant dimensions also presents an unresolved engineering challenge.

#### Stress-relaxation/viscoelastic materials

Viscoelasticity, the time-dependent mechanical behavior inherent to native CNS tissue, has emerged as a distinct biophysical parameter that can be engineered independently of stiffness. A static–dynamic dual-network HyA–collagen hydrogel system allowed tuning of stress relaxation rate (*τ*_1/2_ from ~60 to ~1,200 s) while maintaining constant elastic modulus [92]. Faster relaxation promoted superior neurite extension, neurogenesis, and synapse formation through a mechanism in which matrix stress dissipation reduces myosin-driven retrograde actin flow, prolongs integrin clutch engagement, and decreases Piezo1 expression, ultimately retaining YAP in the cytoplasm [92].

The independent regulation of viscoelasticity and topographical cues was achieved using an aldehyde–methacrylate–hyaluronan/collagen composite hydrogel system [93]. Through a combination of dynamic imine cross-linking and microfluidic coaxial spinning, this system demonstrated that viscoelasticity and nanotopography can be orthogonally tuned to synergistically promote neurite extension and neuronal differentiation [93]. Extending this principle to 3D bioprinted constructs, a GelMA/oxidized HyA (OHA) dynamic covalent network functionalized with N-cadherin (HAVDI) and BDNF (RGI) mimetic peptides synergistically enhanced YAP-mediated mechanotransduction in NSCs through the combination of rapid stress relaxation and specific cell–cell interaction cues, accelerating the self-organization of NSCs into functional neural networks [46].

Dynamic network chemistry can simultaneously govern viscoelasticity and enable injectability with self-healing [94]. Hydrogels cross-linked by reversible Zn^2+^–bisphosphonate coordination bonds undergo network rupture under shear and reformation under static conditions, enabling conformal lesion filling [95]. Similarly, 3,4-dihydroxyphenylalanine (DOPA)-grafted chitosan/designer peptide dual-network hydrogels combine shear-thinning injectability and rapid self-healing through π–π stacking with tissue adhesion and sustained neurotrophin-3 (NT-3) release [96]. A fluorenylmethyloxycarbonyl (Fmoc)-functionalized chitosan/peptide hybrid hydrogel exploits analogous π–π stacking interactions to achieve self-healing while sustaining curcumin release over 7 d with tunable kinetics [97].

Although viscoelastic materials have demonstrated substantial promise, the independent tuning of stress relaxation from stiffness and other biophysical parameters remains technically demanding. Moreover, the long-term safety of chemically dynamic networks, particularly those using synthetic cross-linkers or metal ions, requires thorough evaluation given the sensitivity of regenerating neural tissue. Translation is further complicated by the complex rheological characterization required for quality control in a clinical manufacturing setting.

### Bioelectrical cue-utilizing materials

The native spinal cord functions as an electromechanical system in which electrical signals are integral to neuronal communication and regeneration. Introducing electrical conductivity or piezoelectricity into scaffolds simulates this electroactive microenvironment.

Conductive composite fibers incorporating carbon nanotubes (CNTs) into GelMA exhibit aligned topography and conductivity that promote neuronal differentiation of NSCs. When combined with electrical stimulation, these fibers markedly enhance axonal regeneration, myelination, and functional recovery, restoring tissue conductivity to approximately 1.50 × 10^−3^ S/cm, approaching that of normal neural tissue (~2.13 × 10^−3^ S/cm) [98]. The therapeutic benefit of conductive scaffolds can be further amplified by integrating multiple functionalities. A polycitrate-based nanocomposite hydrogel incorporating polydopamine-coated multiwalled CNTs (PMEACs) simultaneously provides conductivity (0.15 S/m; within the spinal cord range of 0.02 to 0.60 S/m), tissue-matching mechanics (*G′* ~ 291 Pa), injectability, self-healing, tissue adhesion, and broad-spectrum antibacterial activity, promoting axonal regeneration, myelination, and motor recovery in a rat complete transection model [99].

Nanocomposite strategies can also achieve functional integration through simpler formulations. MoS_2_/graphene oxide (GO)/polyvinyl alcohol (PVA) hydrogels prepared by freeze-thaw physical cross-linking leverage the semiconductor properties of MoS_2_ and the conductivity of GO to achieve 0.220 S/m while simultaneously providing peroxidase-like catalytic activity for ROS scavenging [100]. The incorporation of nanosheets unexpectedly reduced the compressive modulus to 220 Pa, closer to the optimal range for neural differentiation, and promoted neuronal differentiation, suppressed astrocytic differentiation, and facilitated M2 macrophage polarization [100].

Moving beyond passive conductivity, piezoelectric materials coupled with ultrasound provide a self-powered, wireless stimulation strategy. Potassium sodium niobate (KNN) piezoelectric nanoparticles embedded in a DSC matrix/GelMA hydrogel generate electrical potentials of approximately 200 to 1,000 mV under ultrasound stimulation at 0.4 W/cm^2^ [101]. This wireless electrical stimulation drives Ca^2+^ influx through voltage-gated calcium channels, activating a CaMKIIb–peroxisome-proliferator-activated receptor γ coactivator 1α (PGC-1α) signaling axis that enhances mitochondrial bioenergetics and adenosine triphosphate synthesis, ultimately promoting NSC neuronal differentiation, endogenous NSC recruitment, and angiogenesis [101].

Despite these advances, bioelectrical materials face distinct translational hurdles. Conductive nanomaterials such as CNTs raise unresolved concerns regarding long-term biodistribution, clearance, and potential cytotoxicity [98,99]. Piezoelectric strategies that rely on external ultrasound stimulation introduce dependency on patient compliance and equipment availability, complicating standardization [101]. Furthermore, the long-term electrochemical stability of these materials in the corrosive and dynamic in vivo environment remains incompletely characterized.

### Biochemically programmed microenvironment

#### Immunomodulatory materials

The immunomodulatory capacity of biomaterials can be programmed through the presentation of specific ECM components. Comparative analysis of primary astrocyte-derived ECM from protoplasmic versus fibrous phenotypes revealed that protoplasmic astrocyte ECM incorporated into HyA hydrogels significantly reduced macrophage and microglial infiltration, decreased CSPG deposition, and promoted axonal growth in a rat dorsal hemisection model, whereas fibrous astrocyte ECM partially reversed the beneficial effects of HyA alone [102].

At the transcriptional and proteomic level, biodegradable polyurethane-DSC ECM composite scaffolds (BWPU-dECM) with an optimized 50:50 ratio were shown to down-regulate 173 proinflammatory genes and up-regulate 19 anti-inflammatory genes while activating the neuroprotective PI3K–AKT pathway at both transcriptional and protein levels, promoting oligodendrocyte differentiation, axonal regeneration, and myelination [103].

The convergence of dynamic network chemistry with ion-mediated signaling offers an additional immunomodulatory strategy. Alginate hydrogels cross-linked by Zn^2+^–bisphosphonate coordination bonds achieve shear-thinning injection, rapid self-healing, and sustained Zn^2+^ release over 14 d [95]. The released Zn^2+^ up-regulates the zinc finger protein A20, a negative regulator of NF-κB, suppressing proinflammatory cytokine expression and promoting M2 macrophage polarization, ultimately facilitating axonal regeneration, myelination, and synapse formation [95].

Despite these promising immunomodulatory strategies, several challenges remain. The use of xenogeneic ECM components raises unresolved questions regarding longer-term immunogenicity and systemic safety [102]. Zn^2+^-mediated immunomodulation, while elegant, requires precise control over release kinetics, as excessive zinc concentrations may be neurotoxic. Furthermore, the long-term stability of immunomodulatory signals in the chronically inflamed lesion microenvironment has not been fully established, and the potential risks associated with pathogen or endotoxin contamination in ECM derived from stem cell sources remain largely unaddressed.

#### Cell delivery materials

Cell delivery strategies require careful matching of material mechanical properties to the specific therapeutic cell population. The SHIELD hydrogel system, a shear-thinning injectable hydrogel composed of a C7 recombinant protein containing multiple adhesive ligands and a polyethylene glycol (PEG)–P1 (proline-rich peptide)–poly(*N*-isopropylacrylamide) (PNIPAM) copolymer, exemplifies this principle [41]. The softest formulation (*G*′ ~ 10 Pa) functionalized with RGDs was optimal for the survival, neurite extension, and consistent engraftment of human induced pluripotent stem cell (iPSC)-derived deep cortical neurons in a rat cervical contusion model, whereas slightly stiffer formulations (*G*′ ~ 160 Pa) were preferred by Schwann cells, highlighting the necessity of tailoring the mechanical microenvironment to the therapeutic cargo [41].

Supporting this concept, a soft injectable salmon fibrin–HyA–laminin hydrogel (*G*′ ~ 200 to 250 Pa) supported human NSC survival and proliferation, while coculture with endothelial colony-forming cells revealed active recruitment and vascular network formation via paracrine secretion of vascular endothelial growth factor (VEGF), platelet-derived growth factor, and TGF-β [104]. The combination of cell delivery with topographical and biochemical cues can further enhance outcomes. N-cadherin-functionalized AFGNs promoted long-term retention and neuronal differentiation of transplanted NSCs in a complete transection model [91].

A central limitation of cell delivery materials is the temporal mismatch between the mechanical requirements of different phases of therapy. The optimal stiffness for acute cell survival and engraftment may differ substantially from the mechanical environment needed to guide long-term differentiation and functional integration. In addition, the survival rate of transplanted cells in the hostile injury microenvironment remains low across all current systems, and the scalability of autologous or iPSC-derived cell sourcing under Good Manufacturing Practice conditions presents a formidable translational barrier.

#### Smart responsive and controlled release materials

Spatiotemporal control over therapeutic delivery can be encoded into scaffold architecture. The coaxial 3D printed scaffold with a gelatin/cellulose nanofiber shell encapsulating MnTBAP and an HyA dual-network hydrogel core demonstrates phase-separated temporal functionality [42]. The shell rapidly degrades within minutes at 37 °C to release MnTBAP, shielding endogenous NSCs from acute oxidative stress, while the core remains stable for over 60 d, providing sustained topographical and N-cadherin-mediated signals [42].

Dynamic network chemistries enable injectable systems that respond to the mechanical demands of the injury site while providing controlled therapeutic release. DOPA-grafted chitosan/designer peptide dual-network hydrogels combine shear-thinning injectability and rapid self-healing with tissue adhesion and sustained NT-3 release, enabling seamless host–graft integration while providing neurotrophic stimulation [96]. Fmoc-functionalized chitosan/peptide hybrid hydrogels exploit π–π stacking for self-healing while sustaining curcumin release over 7 d with release kinetics tunable via network cross-linking density, promoting M2 macrophage polarization and endogenous Schwann-cell-mediated remyelination [97].

Growth factor delivery can also be electrostatically programmed. Heparin–methacrylate incorporated into a 3D printed GelMA hydrogel shell enables sustained electrostatic adsorption and release of basic fibroblast growth factor (bFGF) over 30 d, complementing the topographical and biochemical cues provided by the encapsulated DSC matrix [38]. Similarly, piezoelectric KNN nanoparticles embedded in DSC matrix/GelMA hydrogels respond to external ultrasound stimulation to generate wireless electrical signals, representing a stimuli-responsive strategy in which the therapeutic cue is delivered on-demand rather than through passive release [101].

Although smart responsive systems offer elegant spatiotemporal control over therapeutic delivery, several translational hurdles persist. The multicomponent architecture of many responsive scaffolds increases manufacturing complexity and cost, posing challenges for large-scale production under Good Manufacturing Practice conditions. Externally triggered systems, such as ultrasound-responsive piezoelectric materials, introduce dependency on patient compliance and specialized equipment, limiting applicability in resource-constrained settings. Moreover, the release duration achieved in preclinical models often falls short of the prolonged chronic phase of human SCI recovery, raising questions about whether repeated dosing or implant replacement would be required.

## Future Directions and Challenges

### Deepening of mechanism research

The development of effective mechanotherapeutic strategies for SCI is critically limited by fundamental gaps in the mechanistic understanding of how cells perceive and respond to the dynamic mechanical cues within the injury niche. A primary challenge is the deconvolution of intricate intracellular signaling networks activated by specific mechanical inputs [105]. While pathways such as YAP/TAZ, integrin–FAK, and RhoA/ROCK are frequently implicated, their precise activation thresholds, temporal dynamics, and cell-type-specific downstream effectors remain poorly defined [43]. In particular, which transcriptional cofactors engage YAP under distinct mechanical conditions and whether this binding preference is dictated by stiffness, matrix dynamics, or cell type remains to be experimentally determined. This complexity is compounded by extensive cross-talk between these mechanical cascades and canonical biochemical pathways such as Wnt/β-catenin or inflammatory NF-κB signaling, creating a regulatory network whose logic in dictating cell fate requires systematic elucidation [48,50,61].

Beyond presequenced degradation and release profiles, a major translational hurdle stems from the difficulty of faithfully modeling the multidimensional and dynamic nature of the in vivo mechanical microenvironment. The post-SCI milieu is characterized by a spatially and temporally evolving confluence of stiffness, viscoelasticity, and topographical cues [90]. Current models often examine parameters in isolation, failing to capture critical synergistic interactions [106]. For instance, whether the peptide coating and topographical alignment produce additive or synergistic effects on neuronal differentiation, and how these interactions depend on substrate stiffness, has not been systematically evaluated [56]. Separately, the temporal evolution of these mechanical cues is rarely recapitulated in vitro. How the mechanical properties of implanted scaffolds should evolve over time to match the changing demands of the injury microenvironment has not been systematically investigated. Future research must therefore leverage advanced biofabrication to develop dynamically responsive biomimetic platforms, such as stimuli-responsive or temporally stiffening hydrogels, capable of simulating the injury environment’s progression from acute edema to chronic scarring [36,38,46]. These tools are essential for identifying therapeutic windows where mechanical intervention could be most beneficial.

Furthermore, the fidelity of preclinical models themselves limits mechanistic discovery. Many findings are based on simplified 2D cultures or acute ex vivo models, which may not accurately reflect the multicellular pathology of human SCI [107]. A critical future direction involves using higher-fidelity models, including human spinal cord organoids, chronic large animal SCI models, or multicellular 3D coculture systems [108]. Techniques such as live-cell imaging with mechanical biosensors and single-cell multiomics will be crucial for mapping the spatiotemporal activity of mechanosensitive pathways in these systems [109]. Furthermore, research must account for interindividual variability, as genetic background and injury specifics likely create a spectrum of “personalized” mechanical microenvironments requiring adaptable therapeutic strategies [110]. In particular, whether patient-specific mechanical microenvironments predict optimal scaffold stiffness and therapeutic outcomes remains unknown. Addressing these challenges is imperative for evolving from passive biomaterial implants to active systems capable of dynamically reprogramming the post-SCI landscape.

Complementing these experimental approaches, artificial intelligence is emerging as a powerful tool to accelerate mechanistic discovery and bridge the gap between in vitro observation and in vivo prediction. Machine learning models have already enabled single-cell resolution mapping of macrophage polarization across millions of nanotopographies, identifying specific surface patterns that suppress inflammation via ROCK-dependent mechanotransduction [111]. At the molecular level, artificial-intelligence-driven virtual screening is being applied to design isoform-selective Piezo1 modulators with unprecedented precision [112]. By integrating these computational pipelines with high-content experimental data, future studies can systematically decode the regulatory logic of mechanosensitive networks and predict optimal therapeutic windows across the heterogeneous SCI population. However, whether artificial intelligence models can integrate multimodal data to predict optimal scaffold stiffness and intervention timing for individual patients remains unknown.

### The prospects of transformation in treatment strategies

Synthesizing the intervention strategies reviewed above, Table 6 provides a phase-specific framework of potential approaches based on the spatiotemporal evolution of the mechanical microenvironment. Among these, cell-based mechanomodulation strategies offer the potential to harness endogenous repair mechanisms through physical guidance and mechanical priming. However, the optimal priming protocol, including duration, stiffness and mechanical loading regimen, remains undefined. Integrating mechanical modulation with cell-based therapies represents a key direction, such as using superparamagnetic-iron-oxide-nanoparticle-grafted aligned fibers with alternating magnetic fields to enhance neurite outgrowth from dorsal root ganglia [86]. Furthermore, functionalized biomaterial scaffolds provide multifunctional platforms that can deliver structural support, biochemical signals, and electrical cues to synergistically guide axonal regeneration and neuronal differentiation [25]. The development of “smart” scaffolds capable of responsive factor release or combined with electrical stimulation is actively pursued to enhance NSC differentiation and functional integration [101,113].

**Table 6.** Phase-specific mechanical intervention strategies for spinal cord injury: A framework of potential approaches

Phase	Pathological milieu	Therapeutic goal	Potential intervention strategies
**Acute**	Tissue softening, edema, hemorrhage, inflammatory infiltration, blood–spinal cord barrier disruption	Relieve intraspinal pressure, limit secondary injury cascades, preserve neural tissue	Surgical decompression, extracorporeal shock wave therapy
**Subacute**	Stiffness divergence, reactive astrogliosis, CSPG deposition, peak inflammatory response	Modulate inflammation, inhibit aberrant scar formation, disrupt self-reinforcing gliosis-stiffening loop	Immunomodulatory biomaterials, soft topographical scaffolds RhoA/Piezo1-targeted strategies
**Chronic**	Fibrotic scar maturation, cystic cavitation, persistent but low-grade inflammation, stabilized mechanical barrier	Remodel or bridge the fibrotic scar, provide permissive substrate for axonal regeneration, promote remyelination and functional reconnection	Mechanically matched/viscoelastic hydrogels, topography-guided materials, conductive/piezoelectric scaffolds, cell delivery with biomaterials

CSPG, chondroitin sulfate proteoglycan.

**Fig. 7.**
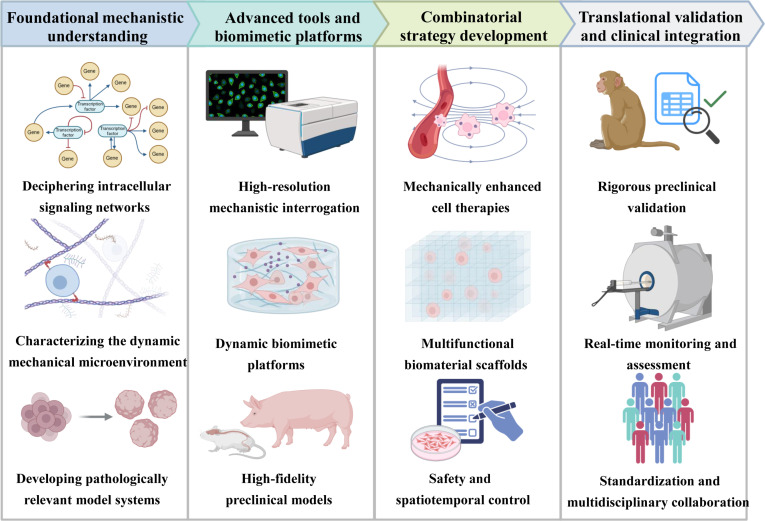
A 4-stage linear translational pipeline for mechano-targeted spinal cord injury (SCI) therapies. This flowchart illustrates the sequential progression from fundamental mechanistic research to clinical integration. (Created with BioRender.com.)

Despite these promising advances, the clinical realization of mechanotargeted strategies faces substantial hurdles, including ensuring long-term biocompatibility of implanted components, achieving precise spatiotemporal control of physical stimuli within heterogeneous spinal cord tissue, and personalizing treatment protocols to accommodate the dynamic evolution of inflammation, fibrosis, and cystic cavitation [114,115]. Bridging this gap from bench to bedside will therefore require a translational pipeline built upon clinically relevant large-animal models [116]. Advanced monitoring technologies will be essential, including diffusion tensor imaging for axonal integrity and optical coherence elastography or ultrasound-based shear wave elastography for noninvasive, real-time mapping of spinal cord mechanical properties during injury progression and therapeutic intervention [117]. Furthermore, sustained multidisciplinary collaboration across bioengineering, materials science, and clinical medicine will be required to optimize biomaterial manufacturing, establish safety standards, and rationally combine mechanobiological interventions with pharmacological agents, rehabilitation training, and cell-based therapies to maximize functional recovery (Fig. 7).

## Conclusion

SCI initiates a profound, dynamic remodeling of the local mechanical microenvironment that evolves in a spatiotemporally heterogeneous manner across acute, subacute, and chronic phases. This process encompasses marked alterations in tissue stiffness, ECM composition, and the presentation of mechanical cues, all of which collectively influence cellular behavior and regenerative outcomes. Key mechanosensitive receptors, including Piezo channels, integrins, and cadherins, transduce these physical signals into biochemical responses, orchestrating critical processes such as neuroglial interactions, immune modulation, and scar formation. The mechanical niche therefore emerges as an active regulator of secondary injury and repair, underscoring its central role in post-SCI pathology.

Emerging therapeutic strategies targeting the mechanical microenvironment show considerable promise for promoting neural repair. Surgical decompression, ESWT, magnetic cell guidance, and advanced biomaterial scaffolds represent innovative approaches aimed at modulating mechanical cues to support neuroregeneration and functional recovery. These interventions act through diverse mechanisms, including alleviating intraspinal pressure, activating mechanotransduction pathways, guiding cell migration, and providing permissive structural support. Biomaterials, in particular, offer versatile platforms for mimicking native tissue mechanics, delivering biochemical signals, and facilitating cellular integration, thereby bridging the lesion site and fostering a regenerative milieu.

Despite these advances, substantial challenges remain in translating mechanobiology insights into clinical practice. A deeper understanding of cell-type-specific mechanosignaling networks, the development of more physiologically relevant injury models, and the establishment of personalized intervention protocols are essential for future progress. Interdisciplinary collaboration across neuroscience, bioengineering, and clinical medicine will be crucial to optimizing therapeutic timing, spatial targeting, and combinatorial strategies. By addressing these translational barriers, mechanotargeted therapies hold strong potential to evolve into effective, precision-based treatments for functional restoration after SCI.
