# Supplementary material for: Mechanical Remodeling and Mechanosensing after Spinal Cord Injury: From Molecular to Translational Approaches
Source: Research (Wash D C). 2026 Jul 10;9:1361. doi: 10.34133/research.1361 (PMC13351121; doi:10.34133/research.1361)
Supplement: Supplementary 1 — Table S1 [file research.1361.f1.docx]

**Tables S1 Extended Data for Key Clinical Trials of Mechanical Interventions for Spinal Cord Injury**

| **Category** | **Study ID*** | **Primary Endpoints** | **Follow-up** | **Major Limitations** |
| --- | --- | --- | --- | --- |
| **Surgical Decompression** | NCT01485458 | ΔASIA motor score at 1 year | 12 months | Underpowered, benefit limited to accelerated recovery. |
|  | NCT04034108 | AIS grade conversion at 6 months | 6 months | Uncontrolled design; individual effects of surgery vs. training cannot be separated. |
|  | NCT04936620 | ΔASIA motor score at 6 months | 12 months | Results pending; includes an ISP/SCPP monitoring sub-study. |
|  | NCT01674764 | WHOQOL-BREF scores at 1 year | 12 months | Baseline injury severity imbalance; QoL influenced by many non-surgical factors. |
|  | NCT05653206 | Feasibility of a core and optional outcome set (ISNCSCI, SF-36, SCIM III, 10m walk, etc.); optional MRI/DTI | Not specified | Not designed to compare intervention efficacy. |
| **Extracorporeal Shock Wave Therapy** | NCT04474106 | Δ Total Motor and Sensory Score (TMSC) at 6 months | 6 months | Narrow therapeutic window (<48h); results pending. |
|  | Comino-Suárez N, et al., 2023^80^ | Ankle passive ROM, gastrocnemius passive resistance, MAS, Penn spasm frequency, SCIM-III, EQ-VAS | 1 week | Single case, short follow-up, no sham control, optimal parameters unknown. |
|  | Li Y, et al., 2022^81^ | NHO size (ultrasound/CT), hip PROM, VAS pain, serum ALP | Not specified | Single case, no control, spontaneous NHO resolution possible, optimal parameters unknown. |
|  | Jeon HM, et al., 2021^82^ | VAS pain, wheelchair sitting tolerance, NHO size (X-ray/CT) | 6 months | Single case, no control, mechanism unclear, optimal parameters unknown. |
|  | Kang N, et al., 2020^83^ | Wound healing (size, granulation), AEs | 3 months | Single case, no control, optimal parameters unknown. |
|  | NCT02203994 | Ashworth Scale (0h, 2h); secondary: Goal Attainment Scale, 10m walk, 6-min walk, etc. | 0-5 days | Results unpublished; single treatment; small sample; optimal parameters unknown. |
| **Scaffolds** | NCT03762655 | AIS grade improvement ≥1 at 6 months | 6 months | Underpowered; unexpectedly high AIS conversion in the control group; sponsor bankruptcy. |
|  | NCT02510365 | Safety and feasibility; ASIA motor/sensory scores | 36-40 months | Uncontrolled design; the effect of scaffold length on therapeutic efficacy remains unclear. |

**Abbreviations:** AEs, adverse events; AIS, ASIA Impairment Scale; ALP, alkaline phosphatase; ASIA, American Spinal Injury Association; CT, computed tomography; DTI, diffusion tensor imaging; EQ-VAS, EuroQol visual analogue scale; ISNCSCI, International Standards for Neurological Classification of Spinal Cord Injury; ISP, intraspinal pressure; MAS, Modified Ashworth Scale; MRI, magnetic resonance imaging; NHO, neurogenic heterotopic ossification; PROM, passive range of motion; QoL, quality of life; SCIM, Spinal Cord Independence Measure; SCPP, spinal cord perfusion pressure; SF-36, 36-Item Short Form Health Survey; TMSC, total motor and sensory score; WHOQOL-BREF, World Health Organization Quality of Life Brief Version.

* Trials were retrieved from ClinicalTrials.gov on April 14, 2026 by searching for “spinal cord injury” as the condition/disease, combined with intervention-related terms including “decompression” “shock wave” “magnetic guidance” “scaffold” and “biomaterial”. Additional keyword searches for "mechanical" "biomechanical" and "stiffness" were performed to identify mechanically relevant studies. Only studies with therapeutic intent, direct relevance to mechanical or mechanobiology-based strategies for SCI, and completed, recruiting, or active status were included. PubMed was additionally searched on the same date for published clinical studies using similar terms.
